# Pancancer outcome prediction via a unified weakly supervised deep learning model

**DOI:** 10.1038/s41392-025-02374-w

**Published:** 2025-09-03

**Authors:** Wei Yuan, Yijiang Chen, Biyue Zhu, Sen Yang, Jiayu Zhang, Ning Mao, Jinxi Xiang, Yuchen Li, Yuanfeng Ji, Xiangde Luo, Kangning Zhang, Xiaohan Xing, Shuo Kang, Dongyuan Xiao, Fang Wang, Jinkun Wu, Haiyan Zhang, Hongping Tang, Himanshu Maurya, German Corredor, Cristian Barrera, Yufei Zhou, Krunal Pandav, Junhan Zhao, Prantesh Jain, Luke Delasos, Junzhou Huang, Kailin Yang, Theodoros N. Teknos, James Lewis, Shlomo Koyfman, Nathan A. Pennell, Kun-Hsing Yu, Xiao Han, Jing Zhang, Xiyue Wang, Anant Madabhushi

**Affiliations:** 1 College of Biomedical Engineering, Sichuan University, Chengdu, Sichuan China; 2 Department of Radiation Oncology, Stanford University School of Medicine, Palo Alto, CA USA; 3 Department of Pharmacy, Children’s Hospital of Chongqing Medical University, Chongqing, China; 4 Department of Radiology, Yantai Yuhuangding Hospital, Qingdao University, Yantai, Shandong China; 5 Department of Pathology, The Affiliated Yantai Yuhuangding Hospital of Qingdao University, Yantai, China; 6 Department of Pathology, Shenzhen Maternity and Child Healthcare Hospital, Futian District, Shenzhen, China; 7 Department of Biomedical Engineering, Emory University, Atlanta, GA USA; 8 Department of Electrical Engineering and Computer Science, Case Western Reserve University, Cleveland, OH USA; 9 Department of Biomedical Informatics, Harvard Medical School, Boston, MA USA; 10 Department of Medical Oncology, Roswell Park Comprehensive Cancer Center, Buffalo, NY USA; 11 Cleveland Clinic Taussig Cancer Center, Cleveland, OH USA; 12 Department of Computer Science and Engineering, The University of Texas at Arlington, Arlington, TX USA; 13 Department of Radiation Oncology, Holden Comprehensive Cancer Center, Iowa Neuroscience Institute, University of Iowa, Iowa City, IA USA; 14 Department of Otolaryngology-Head and Neck Surgery, University Hospitals, Cleveland, OH USA; 15 Department of Pathology, Microbiology and Immunology, Vanderbilt University Medical Center, Nashville, TN USA; 16 Department of Laboratory Medicine and Pathology, Mayo Clinic Arizona, Scottsdale, AZ USA; 17 Department of Radiation Oncology, Cleveland Clinic, Cleveland, OH USA; 18 Department of Pathology, Brigham and Women’s Hospital, Boston, MA USA; 19 Atlanta Veterans Administration Medical Center, Atlanta, GA USA

**Keywords:** Predictive medicine, Outcomes research, Cancer, Medical imaging

## Abstract

Accurate prognosis prediction is essential for guiding cancer treatment and improving patient outcomes. While recent studies have demonstrated the potential of histopathological images in survival analysis, existing models are typically developed in a cancer-specific manner, lack extensive external validation, and often rely on molecular data that are not routinely available in clinical practice. To address these limitations, we present PROGPATH, a unified model capable of integrating histopathological image features with routinely collected clinical variables to achieve pancancer prognosis prediction. PROGPATH employs a weakly supervised deep learning architecture built upon the foundation model for image encoding. Morphological features are aggregated through an attention-guided multiple instance learning module and fused with clinical information via a cross-attention transformer. A router-based classification strategy further refines the prediction performance. PROGPATH was trained on 7999 whole-slide images (WSIs) from 6,670 patients across 15 cancer types, and extensively validated on 17 external cohorts with a total of 7374 WSIs from 4441 patients, covering 12 cancer types from 8 consortia and institutions across three continents. PROGPATH achieved consistently superior performance compared with state-of-the-art multimodal prognosis prediction models. It demonstrated strong generalizability across cancer types and robustness in stratified subgroups, including early- and advanced-stage patients, treatment cohorts (radiotherapy and pharmaceutical therapy), and biomarker-defined subsets. We further provide model interpretability by identifying pathological patterns critical to PROGPATH’s risk predictions, such as the degree of cell differentiation and extent of necrosis. Together, these results highlight the potential of PROGPATH to support pancancer outcome prediction and inform personalized cancer management strategies.

## Introduction

Cancer remains a major global health burden, with an estimated 20 million new cases and 9.7 million cancer-related deaths worldwide in 2022.^1^ Accurate prognosis assessment is essential for evaluating disease progression and guiding treatment decisions. However, traditional prognosis systems, such as tumor-node-metastasis (TNM) staging, have notable limitations in capturing the extensive interpatient heterogeneity observed in cancer.^2,3^ While molecular biomarkers such as microsatellite instability (MSI) in colorectal cancer^4,5^ and human epidermal growth factor receptor 2 status in breast cancer^6,7^ can enhance prognostic accuracy, their routine use is constrained by cost, accessibility, and disease specificity.^8,9^ Therefore, more precise, accessible, and scalable methods of prognostic evaluation are urgently needed to enable personalized treatment planning and improve survival outcomes across diverse cancer types.

Histopathological analysis is the gold standard for cancer diagnosis and is central to cancer prognosis.^10–12^ These tissue samples capture detailed information on tumor morphology and its microenvironment, including angiogenesis, immune infiltration, and stromal composition,^13–16^ all of which are known to affect clinical outcomes. The digitization of histopathology slides, combined with recent advances in artificial intelligence (AI), has opened new avenues for leveraging computational pathology tools as a scalable and cost-efficient solutions for cancer prognosis. By extracting prognostically relevant features directly from whole-slide images (WSIs), AI-powered models offer a promising alternative to expensive and disease-specific molecular assays. Consequently, computational histopathology is rapidly emerging as a viable strategy for survival prediction and risk stratification across diverse cancer types.^17–40^ Existing computational pathology efforts for cancer prognosis can be broadly categorized into histopathology-only approaches,^19–27,40^ and methods that incorporate multimodal or auxiliary information, such as clinical, molecular, or genomic data alongside histopathological images.^17,18,28–39^

Owing to the extremely large size of WSIs, computational pathology approaches typically divide them into smaller image tiles and then aggregate tile-level information to enable slide-level analysis.^19–27,40^ Multimodal approaches for survival prediction typically integrate histopathological images with additional clinical, molecular, or genomic data.^17,18,28–39^ Some studies have combined image-derived features with clinical features such as tumor grade,^28,39^ tumor stage,^28,38^ histological subtypes,^39^ or demographics^33^ to predict patient outcomes. Several other studies have incorporated genomic or molecular profiles alongside histopathological features to improve prognosis prediction.^29–37^ A few studies have attempted pancancer survival modeling, which refers to training a single model across multiple cancer types. These studies developed models by combining clinical, molecular, and genomic data with histopathology data from The Cancer Genome Atlas (TCGA) dataset.^17,18^ Pancancer models offer the potential to learn shared prognostic patterns across cancer types, which may improve generalizability and predictive performance.^18^

Despite these promising developments, existing approaches face several critical limitations. First, the majority of prior studies adopt a cancer-specific modeling strategy, in which separate models are trained for each cancer type.^19–37,41^ While this approach could be effective within single cancer cohorts, it hinders generalizability and scalability. Notably, in the limited pancancer studies available, incorporating histopathological features did not yield performance gains.^17,18^ Second, many studies lack rigorous external validation across diverse cancer types and independent data sets,^17,18,20,22,24,26,27,29–37,39,40^ raising concerns regarding model robustness, reproducibility, and generalizability to real-world clinical settings. Third, although multimodal methods that incorporate genomic data alongside histopathology have demonstrated strong performance,^29–32,34–37^ they often rely on molecular assays that are expensive, time-consuming, and not routinely available in many clinical workflows, limiting their practical deployment.

To address the aforementioned gaps in the field, we present PROGPATH, a unified pancancer prognosis prediction model that integrates histopathological image features with routinely collected clinical variables, such as age, sex, and tumor stage. Rather than analyzing every image region equally, PROGPATH learns to focus on the most informative areas within each slide by assigning varying levels of importance, mimicking how pathologists prioritize regions during manual review. These region-level patterns are then aggregated to produce a comprehensive slide-level representation. To further improve accuracy, PROGPATH combines visual and clinical information through a feature matching mechanism that models the relationship between the two data modalities. Finally, instead of using a single static decision pathway, the model incorporates a flexible strategy that selects the most appropriate subtype-specific predictors, allowing it to adapt its decision-making across cancer types. PROGPATH was developed using 7999 slides from 6670 patients, including 15 cancer types, and it has been extensively validated on 17 external cohorts with a total of 7374 WSIs from 4441 patients, covering 12 cancer types across eight consortia and institutions spanning three continents. PROGPATH consistently outperformed existing state-of-the-art approaches as well as single-modality baselines (using only histopathological images or clinical variables).

## Results

### Overview architecture of PROGPATH

**Fig. 1:**
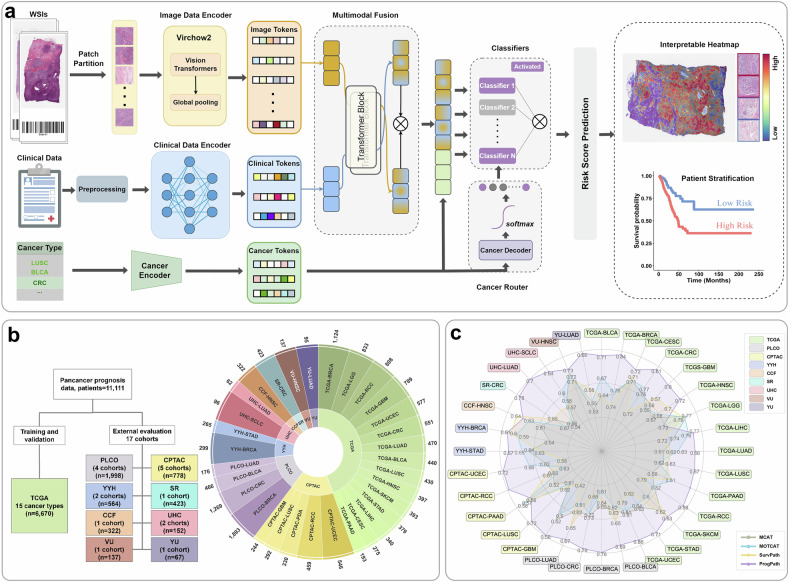
Overview of the prognostic prediction model (PROGPATH). **a** Workflows of PROGPATH. The input consists of digitized hematoxylin and eosin (H&E) slides, corresponding clinical variables, and cancer type information. Patch-level features are extracted from WSIs using Virchow2—a Vision Transformer-based foundation model pretrained for computational pathology. Clinical and cancer-type features are embedded via two independent fully connected networks. The image and clinical data representations are fused using a cross-attention transformer to model intermodal interactions. The fused representations are further concatenated with cancer-specific tokens, which are decoded into adaptive activation weights for a cancer-aware router. This router dynamically selects among domain-specific classifiers, enabling the generation of accurate and cancer-tailored survival predictions. **b** Data distribution. PROGPATH was developed using the TCGA cohorts (*n* = 6670) and has been extensively evaluated on 17 independent datasets (*n* = 42441), covering total of 15 cancer types. *n* represents the number of patients. **c** Overall performance in terms of the concordance index (C-index). PROGPATH significantly outperforms other methods across 32 datasets comprising 15,373 slides

The overview architecture of our PROGPATH is illustrated in Fig. 1, which represents a unified model for pancancer survival prediction. PROGPATH first leverages standard image preprocessing techniques, including tiling and patch-level feature extraction, via the foundation model Virchow2.^42,43^. These features are then aggregated via a deep attention model,^44^ followed by a cross-attention transformer module that integrates histopathological and clinical features to produce patient-level survival predictions. To further enhance pancancer prognosis analysis, we incorporate a cancer-aware router that dynamically selects domain-specific classifiers (Fig. 1a).

Model training was conducted using the TCGA cohorts,^45^ comprising 7999 WSIs from 6670 patients across 15 cancer types. PROGPATH is trained via a 5-fold cross-validation approach. To evaluate the generalizability of PROGPATH, we conducted extensive external validation on 17 independent cohorts drawn from 8 consortia and institutions, comprising 7374 WSIs from 4441 patients across 12 tumor types. These included established public resources, such as the Prostate, Lung, Colorectal and Ovarian (PLCO)^46,47^ and the Clinical Proteomic Tumor Analysis Consortium (CPTAC),^48^ as well as six international institutions: Yantai Yuhuangding Hospital (YYH), Cleveland Clinic Foundation (CCF), University of St Andrews (SR),^49^ University Hospitals Cleveland (UHC), Vanderbilt University (VU), and Yale University (YU) (Fig. 1b). Owing to their longstanding roles in cancer epidemiology and established use as benchmarking datasets, PLCO and CPTAC are referred to as external benchmark cohorts. The remaining eight external cohorts are institution-specific datasets, comprising one newly released public dataset and seven private institutional datasets, highlighting their multicenter origin and clinical heterogeneity. Furthermore, we benchmarked PROGPATH against three recent state-of-the-art multimodal survival prediction models: MCAT,^30^ MOTCAT,^31^ and SurvPath^32^ (Fig. 1c). This comparison underscores the performance advantage and modeling advances introduced by our approach.

Survival outcomes were defined on the basis of the availability and reliability of endpoint annotations. Disease-specific survival (DSS) was used for the TCGA, PLCO, and SR cohorts, reflecting its relevance for cancer-related mortality. Overall survival (OS) was adopted for the CPTAC, CCF, UHC, and YU datasets because of limited cause-of-death annotations. The YYH dataset includes OS and disease-free interval (DFI) information. Detailed information is shown in Supplementary Table 1.

### Integrating histopathology and clinical features enhances prognostic prediction

**Fig. 2:**
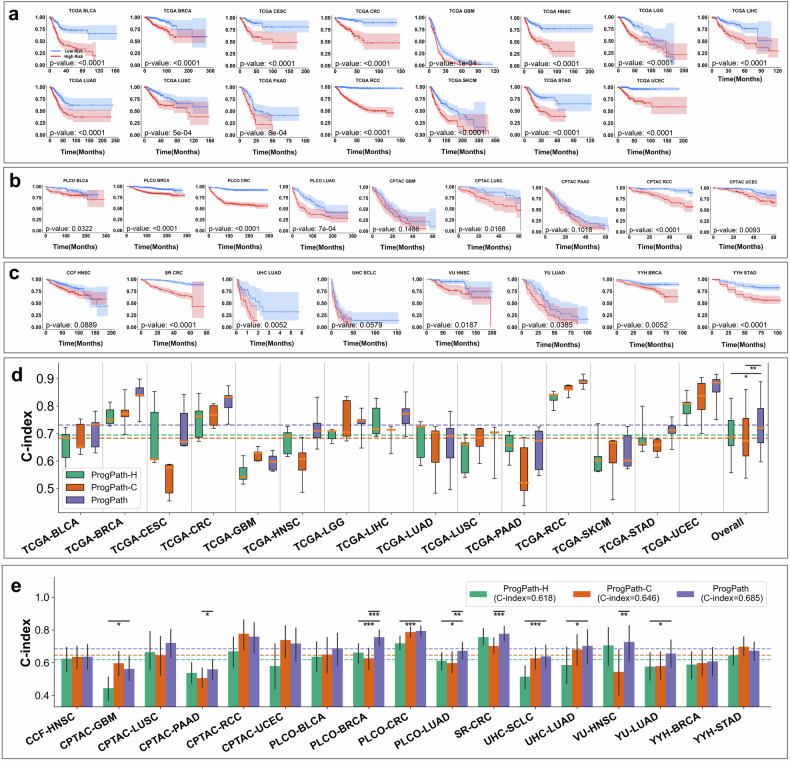
Performance of PROGPATH. Comparison of PROGPATH-H (histopathology only), PROGPATH-C (clinical features only), and the integrated PROGPATH model. All Kaplan–Meier curves are plotted with 95% confidence intervals (CIs). **a** Kaplan–Meier curves of PROGPATH in the TCGA cohorts, with clear separation confidence intervals between risk groups in 9 out of 15 cohorts. **b** Kaplan–Meier curves for the PLCO and CPTAC external cohorts, with clear separation confidence intervals between risk groups in five out of nine cohorts. **c** Kaplan–Meier curves in independent clinical cohorts, with clear separation confidence intervals between risk groups in 4 out of 8 cohorts. **d** C-index box plots across 15 cancer types in the TCGA held-out test sets. The integrated PROGPATH significantly outperforms PROGPATH-H and PROGPATH-C, with relative improvements of 5.3 and 7.0%, respectively. The overall box plots summarize the distribution of model performance (mean and standard deviation) across cancer types, with statistical significance indicated. P values were calculated via the Mann-Whitney U test on the basis of the mean 5-fold performance of each method. **e** Bar plots of C-index scores in external validation cohorts (PLCO, CPTAC, and independent clinical datasets) with significant markers. PROGPATH achieves average improvements of 8.5 and 5.5% over PROGPATH-H and PROGPATH-C, respectively. The error bars represent 95% confidence intervals estimated via the bootstrap method with 1000 replicates. P values were calculated using a two-sided z test on the basis of the bootstrap samples. In **d** and **e**, dashed horizontal lines in both panels indicate the average C-index per model across all cancer types. Significant markers are defined as follows: * for *p* < 0.05, ** for *p* < 0.01, and *** for *p* < 0.001

Clinical variables such as age, sex, and tumor stage were integrated with histopathological features via a cross-attention transformer to construct an integrated prognostic model (PROGPATH). To assess the prognostic performance of PROGPATH, we compared PROGPATH against two unimodal baselines: PROGPATH-H, which uses only histopathology image data, and PROGPATH-C, which relies solely on clinical features. Both PROGPATH-H and PROGPATH-C employed our unified pancancer training strategy, cancer-type encoding, and the router mechanism. They were trained and validated on the same TCGA data and subsequently tested on 17 independent external cohorts (Fig. 2, Supplementary Table 2). To benchmark PROGPATH against the standard clinical approach for survival prediction, we further compared it to Cox proportional hazards models,^50^ which were constructed using the same routinely available clinical variables (age, sex, and tumor stage) (Supplementary Fig. 1, Supplementary Table 3). This direct comparison highlights the added prognostic value of PROGPATH over traditional statistical methods.

In the TCGA held-out test sets (Fig. 2d, Supplementary Table 2), PROGPATH achieved a mean concordance index (C-index) of 0.731, outperforming PROGPATH-H (0.694) and PROGPATH-C (0.683) by 5.3 and 7.0%, respectively. In terms of the mean area under the curve (AUC) metric, PROGPATH reached 0.737, significantly exceeding the performance of PROGPATH-H (0.706) and PROGPATH-C (0.674). Kaplan–Meier analyses (Fig. 2a) further demonstrated that PROGPATH successfully stratified all cohorts into high- and low-risk groups (log-rank *p* < 0.05), in which bladder urothelial carcinoma (BLCA), cervical squamous cell carcinoma and endocervical adenocarcinoma (CESC), colorectal adenocarcinoma (CRC), head and neck squamous cell carcinoma (HNSC), lung adenocarcinoma (LUAD), pancreatic adenocarcinoma (PAAD), renal cell carcinoma (RCC), stomach adenocarcinoma (STAD), and uterine corpus endometrial carcinoma (UCEC) cancer types presented clear risk separations in confidence intervals.

We further validated PROGPATH across a broad range of external datasets (Fig. 2e, Supplementary Table 2). In the PLCO cohorts, PROGPATH achieved a mean C-index of 0.727, whereas it was 0.657 for PROGPATH-H and 0.665 for PROGPATH-C. The improvements in both the C-index and time-dependent AUC metrics were consistent across BLCA, breast invasive carcinoma (BRCA), CRC, and LUAD. In the CPTAC cohorts, PROGPATH achieved a C-index of 0.664, outperforming PROGPATH-H (0.579) and PROGPATH-C (0.652). In the independent clinical cohorts, which include a recently released public dataset along with 7 institutional private datasets, PROGPATH achieved a mean C-index of 0.677, surpassing both PROGPATH-H (0.624) and PROGPATH-C (0.633). Across all the external cohorts, clear separation confidence intervals were observed in 9/17 cohorts (Fig. 2b, c). Although partial overlap of 95% confidence intervals was observed at later time points in some cohorts, this likely reflects smaller numbers of patients at risk as follow-up progresses.^51^ The trends in the time-dependent AUCs were consistent with those of the C-indices but presented relatively high values (Supplementary Table 2).

### PROGPATH outperforms state-of-the-art baselines

**Fig. 3:**
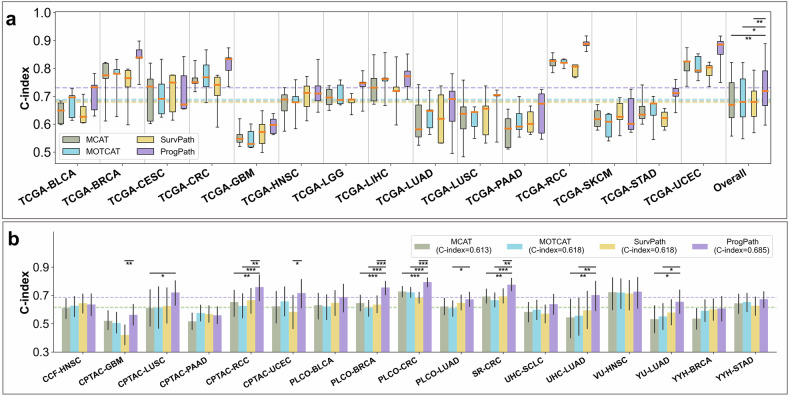
Comparative performance of PROGPATH and state-of-the-art multimodal baselines. Comparison of PROGPATH with three representative multimodal survival prediction models: MCAT, MOTCAT, and SurvPath. **a** C-index comparison via box plots across 15 cancer types in the TCGA held-out test sets. PROGPATH outperforms MCAT, MOTCAT, and SurvPath across the board. The overall box plots summarize the distribution of model performance (mean and standard deviation) across cancer types, with statistical significance indicated. P values were calculated via the Mann-Whitney U test on the basis of the mean 5-fold performance of each method. **b** Bar plots of C-index performance in external cohorts with significant markers. PROGPATH outperforms MCAT, MOTCAT, and SurvPath across the cancer types. The error bars represent 95% confidence intervals estimated via the bootstrap method with 1000 replicates. P values were calculated via a two-sided z test on the basis of the bootstrap samples. In **a** and **b**, the dashed horizontal line for each model represents the average C-index across the analyzed cancer types. Significant markers are defined as follows: * for *p* < 0.05, ** for *p* < 0.01, and *** for *p* < 0.001

We compared PROGPATH with three leading multimodal survival prediction models—MCAT,^30^ MOTCAT,^31^ and SurvPath^32^—using identical histopathology and clinical inputs to ensure a fair evaluation (Fig. 3, Supplementary Table 4).

In the TCGA cohorts, all the models achieved competitive performance (Fig. 3a, Supplementary Table 4). PROGPATH achieved a mean C-index of 0.731, surpassing those of MCAT (0.683), MOTCAT (0.689), and SurvPath (0.678) by 7.0, 6.1, and 7.8%, respectively. With respect to the AUC metric, PROGPATH also outperformed these models, achieving 0.737 compared with 0.678 (MCAT), 0.688 (MOTCAT), and 0.689 (SurvPath), representing relative improvements of 8.7, 7.1, and 7.0%, respectively. PROGPATH outperformed all the baselines in 14 of the 15 cancer types (Fig. 3a), with the exception of skin cutaneous melanoma (SKCM), where the performance difference was negligible (C-index difference*<*0.001).

More substantial performance gains were observed in the external validation (Fig. 3b, Supplementary Table 4). In the PLCO cohorts, PROGPATH reached a C-index gains of 10.8, 13.6, and 11.2% over MCAT, MOTCAT, and SurvPath, respectively. In the CPTAC cohort, the corresponding improvements were 13.5, 11.4, and 15.9%, respectively. Across the independent clinical cohorts, PROGPATH demonstrated a consistent advantage, surpassing the next-best model, SurvPath, by an average C-index of 7.7%.

Although PROGPATH achieved higher mean C-index scores than existing baseline models did in 15 out of 17 benchmark datasets, statistically significant improvements (*p* < 0.05, two-sided z test) were observed in 10 cohorts (Fig. 3b, Supplementary Table 4). PROGPATH showed modest underperformance in the CPTAC-PAAD and CCF-HNSC cohorts. In PLCO-BLCA, UHC-SCLC, VU-HNSC, YYH-BRCA, and YYH-STAD, while the mean C-index was greater, the differences were not statistically significant. We hypothesize that this may be attributed to the model’s unified pancancer design, which prioritizes learning shared prognostic patterns across cancer types, potentially at the expense of cohort-specific optimization.

Moreover, we present a direct comparison of our approach with PORPOISE,^29^ MCAT,^30^ MOTCAT,^31^ and SurvPath,^32^ using shared cohorts from the TCGA consortia (Supplementary Table 5). Overall, PROGPATH achieved the best performance in most cancer types. The exception was lower grade glioma (LGG), where PORPOISE’s focus on gene mutations, such as the *IDH1* mutation status^29^ provided an advantage, whereas PROGPATH utilized only age and sex information, because LGG does not use a conventional staging system. Notably, the four compared approaches exhibit three key constraints: (1) cancer-specific approach: all methods need to train separate models for each cancer type; (2) narrow scope: most of these studies (MCAT, MOTCAT, and SurvPath) focus on limited cancer types (≤5), therefore lacking insight for pancancer analysis; and (3) validation gaps: none of these methods provide external validation to investigate their generalizability across heterogeneous clinical settings.

These consistent and significant performance gains across both in-distribution and out-of-distribution datasets demonstrate that PROGPATH not only achieves state-of-the-art accuracy but also exhibits strong generalizability. This robustness is important for practical applications in clinical settings.

### Robust performance across cancer stages

Given the clinical importance of stage-specific risk assessment in treatment planning, we further evaluated the performance of PROGPATH-H, PROGPATH-C, and PROGPATH in stratifying patient outcomes across early-stage (stages I–II) and advanced-stage (stages III–IV) cancers (Supplementary Fig. 2–7).

For early-stage patients in the TCGA held-out test sets, PROGPATH achieved statistically significant stratification (*p* < 0.05 in log-rank tests) across all 12 evaluated cancer types (Supplementary Fig. 2). PROGPATH achieved an overall C-index of 0.728, compared with 0.706 for PROGPATH-H and 0.628 for PROGPATH-C, representing relative gains of 3.1 and 15.9%, respectively.

External validation of PROGPATH in the PLCO, CPTAC, and independent clinical cohorts yielded C-indices of 0.643, 0.661, and 0.654, respectively (Supplementary Fig. 3). The most substantial improvement was observed in the CPTAC-LUSC early-stage cohort, where PROGPATH outperformed PROGPATH-C with a 17.2% relative increase in the C-index and a 19.8% increase in the time-dependent AUC.

We further evaluated the performance of PROGPATH for advanced-stage cancers (stage III-IV) (Supplementary Fig. 4-5) and for stage III cancers (Supplementary Fig. 6-7). In both settings, PROGPATH generally outperformed PROGPATH-H and PROGPATH-C, with statistically significant risk stratification observed across most cancer types on the basis of Kaplan–Meier analysis. These results highlight the robustness of the PROGPATH framework in handling multisource data for the prediction of outcomes in various stages of the disease.

### Stratification across treatment subgroups

**Fig. 4:**
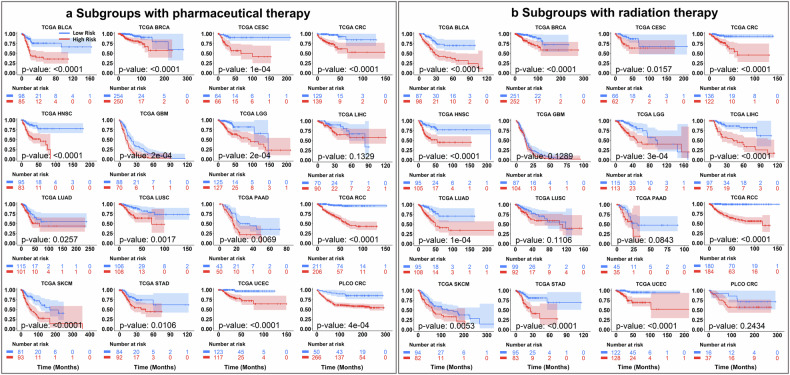
Kaplan–Meier survival analysis of treatment-specific subgroups stratified by PROGPATH score. Survival stratification performance of PROGPATH for patients receiving pharmaceutical therapy or radiotherapy in the TCGA cohorts and the PLCO-CRC dataset. All Kaplan–Meier curves are plotted with 95% confidence intervals (CIs). **a** Kaplan–Meier curves for subgroups receiving pharmaceutical therapy. PROGPATH can distinguish between low- and high-risk patients in 15 out of 16 evaluated subgroups (*p* < 0.05). **b** Kaplan–Meier curves for subgroups treated with radiotherapy. PROGPATH can distinguish between low- and high-risk patients in 12 out of 16 evaluated subgroups (*p* < 0.05)

In clinical practice, treatment decisions such as pharmacotherapy and radiotherapy can result in substantial variability in patient survival outcomes.^52–54^ To evaluate whether PROGPATH can capture treatment-associated prognostic differences, we assessed its ability to stratify patients receiving pharmacologic or radiotherapeutic interventions (Fig. 4).

Across 15 cancer types treated with pharmacotherapy, PROGPATH significantly stratified high-risk and low-risk patients in 15 subgroups, as confirmed by log-rank tests (*p* < 0.05; Fig. 4a). Notably, clear separation confidence intervals between risk groups were observed in seven out of 16 cohorts. A similar pattern was observed in the radiotherapy subgroup, with significant stratification achieved in 12 subgroups (Fig. 4b). Notably, clear separation confidence intervals between risk groups were observed in seven out of 16 cohorts. These findings highlight the robustness of PROGPATH in capturing treatment-associated prognostic differences and suggest that its predictions reflect clinically meaningful variations in therapeutic response.

### Performance across biomarker-defined subgroups

**Fig. 5:**
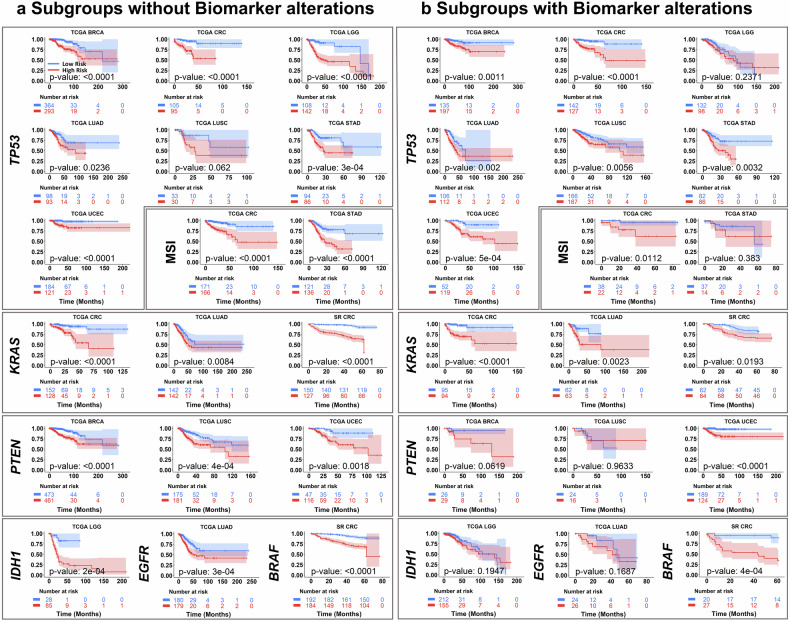
Kaplan–Meier analysis of patient subgroups stratified by PROGPATH score based on the basis of biomarker status. Stratification performance of PROGPATH in patients with or without common biomarker alterations. All Kaplan–Meier curves are plotted with 95% confidence intervals (CIs). **a** Subgroup analysis of patients without specific biomarker alterations. PROGPATH successfully stratifies low- and high-risk patients in 17 out of 18 evaluated subgroups (*p* < 0.05). **b** Subgroup analysis of patients with specific biomarker alterations. PROGPATH can distinguish between low- and high-risk patients in 12 out of 18 evaluated subgroups (*p* < 0.05)

Given the growing role of genomic biomarkers in guiding cancer prognosis and treatment, we assessed whether PROGPATH provides complementary prognostic value within biomarker-defined patient subgroups. We further evaluated the prognostic utility of PROGPATH in patient subgroups stratified by biomarker status. Seven commonly studied cancer biomarkers were selected: *TP53*, *PTEN*, *KRAS*, MSI, *IDH1*, *EGFR*, and *BRAF*. The results of the Kaplan–Meier analyses for patients without and with specific biomarker alterations are presented in Fig. 5a and Fig. 5b, respectively.

Among biomarker-negative patients, PROGPATH successfully stratified survival risk across most cancer types (Fig. 5a), with the exception of the *TP53*-wild type subgroup in LUSC, which did not reach statistical significance. In the biomarker-positive subgroups (Fig. 5b), significant stratification was achieved in 12 of the 18 evaluated cohorts (66.7%), on the basis of the log-rank test (*p* < 0.05). Both the biomarker-negative and biomarker-positive risk groups exhibited clear separation confidence intervals in eight of the 18 evaluated cohorts, further supporting the robustness of the model’s prognostic estimates. In several subgroups, statistical significance was borderline (0.05), likely due to a smaller patient sample size. These results suggest that PROGPATH provides complementary prognostic value beyond genomic biomarkers and may support future biomarker discovery and validation in precision oncology.

## Discussion

In this study, we present PROGPATH, the first unified and generalizable pancancer prognosis prediction framework that integrates histopathological images with routinely collected clinical variables. Built upon a foundation model^42,43^ for image representation and enhanced by a transformer-based fusion mechanism, PROGPATH demonstrates superior prognostic prediction capabilities across diverse cancer types and patient subgroups.

Notably, PROGPATH distinguishes itself from prior pancancer prognosis studies,^17,18^, which have relied primarily on molecular and genomic data that limit broader clinical use due to accessibility constraints. By treating histopathology as the central information source and employing a foundation model for extracting high-level representations, our framework captures significant histological prognostic signals across cancer types. Furthermore, we introduced a novel cancer type-specific routing mechanism to preserve disease-specific discriminative patterns. The model was developed via the TCGA cohorts covering 15 cancer types and extensively validated via a large-scale, geographically diverse set of 17 independent cohorts—including ten public datasets from CPTAC, PLCO, and the University of St Andrews, as well as seven private cohorts from five institutions—totaling 15,373 histopathology slides from 11,111 patients. This rigorous validation underscores PROGPATH’s robustness, generalizability, and cross-cohort applicability, setting a new benchmark in the clinical deployment of AI-driven cancer prognosis.

A universal challenge in patient-level computational pathology analyses lies in analyzing extremely large WSIs. Conventional approaches divide them into smaller tiles and aggregate patient-level signals with multiple instance learning (MIL) frameworks. Recent MIL solutions span from mean-pooling to advanced deep learning methods, such as graph neural networks,^24,27^ cluster-based methods,^19^ ranking-based models,^22^ and more.^19,20,22–24,26,27^ To address this, PROGPATH leverages a foundation model (Virchow2) for rich tile-level encoding, enabling extraction of nuanced morphological features from tissue samples; employs an attention-guided MIL aggregator to focus on the most diagnostically relevant regions rich in information,^22,55^ mimicking a pathologist’s prioritization during visual assessment; and fuses histology and clinical variables via a cross-attention transformer to integrate tissue morphology with patient demographics, akin to an oncologist’s holistic assessment.

We also conducted head-to-head comparisons with existing multimodal deep learning methods for prognosis analysis, including MCAT,^30^ MOTCAT,^31^ and SurvPath.^32^ All the models were trained and evaluated via identical inputs, namely, foundation-model-derived image features and the same clinical data, ensuring a fair comparison of architectural design and modeling strategies. Under a unified pancancer setting, PROGPATH consistently outperformed these baselines. Notably, these previous methods were typically developed for small, cancer-specific cohorts and relied heavily on genomic inputs, limiting their scalability and clinical applicability. In contrast, PROGPATH achieves robust and generalizable prognosis prediction by leveraging widely available clinical variables (age, sex, and stage) alongside histopathological information. This seamless fusion not only increases predictive accuracy but also paves the way for rapid, cost-effective risk stratification in routine pathology workflows—minimizing dependence on specialized molecular assays and accelerating clinical translation.

To further assess the contribution of multimodal integration in PROGPATH, we conducted an ablation study comparing its clinical-only variant (PROGPATH-C) and WSI-only variant (PROGPATH-H), both of which share our unified pancancer training strategy, cancer-type encoding, and the router mechanism. While PROGPATH-C achieves competitive performance in several cohorts, PROGPATH consistently outperforms both single-modality variants on a majority of the external datasets, demonstrating the benefit of integrating histopathology and clinical features. In addition, we compared PROGPATH with traditional Cox proportional hazards models based solely on clinical variables (age, sex, and stage). PROGPATH significantly outperforms the Cox models in 11 out of 17 cohorts (Supplementary Fig. 1 and Supplementary Table 3), underscoring the limitations of conventional approaches and the added value of our deep learning-based framework.

Traditional staging systems (e.g., TNM) capture the extent of an anatomic tumor but often miss subtle microarchitectural cues, losing prognostic accuracy as the stage advances. In contrast, PROGPATH maintains balanced accuracy across early and late-stage patients. However, better prognostic performance alone does not explain why morphology improves risk stratification. To address this, we analyzed model attention heatmaps, which revealed that the model consistently focused on histopathological regions marked by poor differentiation, a high nuclear-cytoplasmic ratio, necrosis, and desmoplastic stroma in high-risk patients. In contrast, low-risk predictions emphasized well-formed glandular structures and dense lymphocytic infiltration (Supplementary Fig. 11). These features are consistent with known biological pathways, such as hypoxia-induced necrosis, immune editing, and cell cycle dysregulation. Together, these findings suggest that PROGPATH captures the prognostic morphology overlooked by TNM, offering both performance gains and mechanistic insight.

PROGPATH also enables stratification within treatment and molecular subgroups where conventional clinical and genomic indicators often fail to fully resolve risk. Across the pharmacotherapy and radiotherapy cohorts in 15 cancer types, PROGPATH achieved significant survival separation in 15 and 12 subgroups, respectively (*p* < 0.05), suggesting that morphological correlates of treatment sensitivity or resistance are embedded in histology. For example, attention maps in poor-outcome cases frequently localize to regions of stromal expansion and necrosis, which may reflect underlying hypoxia-driven resistance mechanisms or immunologically cold microenvironments—features typically underreported in structured pathology or clinical records. Within molecular subgroups defined by mutations in MSI, *EGFR*, *IDH1*, *KRAS*, and *TP53*, PROGPATH stratifies survival in nearly 70% of cohorts, with clear separation of confidence intervals in close to half. These results suggest that while two tumors may share the same mutation status, they can differ significantly in morphology, immune context, and tumor microenvironment. This highlights how PROGPATH can provide complementary information to molecular assays by identifying downstream phenotypes that reflect both genetic alterations and the interaction of a tumor with its environment. These findings point to the potential of AI-based histology to guide prognosis and treatment decisions, especially in settings where genomic testing is unavailable or incomplete.

PROGPATH has demonstrated consistent and generalizable performance across diverse cancer types and multicenter cohorts. By jointly leveraging histopathological images and key clinical variables, the model captures complementary prognostic signals that may not be apparent when clinical features are used alone. This multimodal approach provides nuanced risk stratification, offering valuable insights into patient prognosis. Furthermore, clinical variables alone, although robust, may be limited in the context of narrowly defined subgroups, where small sample sizes prevent meaningful statistical comparisons. In such clinically specific, data-scarce scenarios, integrating histopathological image data, as implemented in PROGPATH, provides additional granularity and enhances prognostic resolution beyond basic demographic and staging variables. However, we emphasize that the current utility of PROGPATH is primarily prognostic rather than predictive. Although a robust prognosis can broadly inform clinical management, our model does not yet demonstrate explicit predictive capabilities to guide specific therapeutic decisions or interventions. Future studies, including prospective validation trials, are necessary to evaluate the predictive potential and actionable clinical utility of PROGPATH, thereby advancing personalized cancer care as digital pathology becomes increasingly integrated into routine oncology practice.

Nevertheless, this study is not without its limitations. First, the clinical variables used are limited to basic demographic and staging information. The incorporation of richer clinical narratives and treatment histories, potentially through advanced natural language processing, may further improve performance. Second, stratifying patients into three risk groups (low, intermediate, and high) could enable more precise predictions. However, the current sample size for some cancer types is insufficient to support refined stratification. Future studies with larger and more balanced datasets may enable this extension. Third, while PROGPATH benefits from large-scale multicancer training, the current version does not account for the effects of emerging therapies, such as immunotherapy or targeted agents. Future studies should integrate treatment-specific outcomes to better guide therapeutic decision-making.

In conclusion, we present PROGPATH, a robust pancancer survival prediction framework that leverages both histopathological images and routinely available clinical data. Through large-scale validation across diverse cancer types and patient populations, PROGPATH demonstrates strong generalizability, outperforming existing unimodal and multimodal approaches. Its consistent performance across clinical stages, treatment contexts, and biomarker-defined subgroups underscores its potential as a reliable prognostic tool. Furthermore, its ability to highlight biologically meaningful morphological patterns contributes to model interpretability and clinical trust. As an open-source tool, PROGPATH lays a scalable foundation for future multimodal AI applications in oncology and may support personalized treatment planning in real-world clinical settings.

## Methods

### Data sources

In this study, we utilized a total of 15,373 standard hematoxylin–eosin (H&E) stained tumor tissue slides, sourced from 11,111 patients across 15 cancer types to predict the prognosis of various cancers. These WSIs were curated from the following cohorts: TCGA,^45^ the CPTAC, the PLCO,^46,47^ YYH, CCF, SR,^49^ UHC, VU, and YU. Clinical information, including age, sex, race, and tumor stage, was also included in these large-scale datasets. The detailed patient characteristics are reported in Supplementary Table 1. The TCGA data were used for model training, and the remaining 17 cohorts from PLCO, CPTAC, YYH, CCF, SR, UHC, VU, and YU were used for the independent validation. Ethics approval was obtained from the institutional review board of participating centers, including the Yantai Yuhuangding Hospital (IRB-2025-630), Cleveland Clinic Foundation (IRB-14-551), University Hospitals Cleveland (IRB-02-13-42C), Vanderbilt University (IRB-151366), and Yale University (IRB-9505008219 and IRB-1608018220). Informed consent was waived for this retrospective analysis.

The TCGA contains a total of 7999 WSIs spanning 15 cancer types, including bladder urothelial carcinoma (BLCA, 440 WSIs), BRCA (BRCA, 1124 WSIs), cervical squamous cell carcinoma and endocervical adenocarcinoma (CESC, 275 WSIs), glioblastoma multiforme (GBM, 789 WSIs), head and neck squamous cell carcinoma (HNSC, 397 WSIs), brain LGG (LGG, 833 WSIs), liver hepatocellular carcinoma (LIHC, 340 WSIs), lung adenocarcinoma (LUAD, 470 WSIs), lung squamous cell carcinoma (LUSC, 430 WSIs), pancreatic adenocarcinoma (PAAD, 193 WSIs), skin cutaneous melanoma (SKCM, 393 WSIs), stomach adenocarcinoma (STAD, 379 WSIs), uterine corpus endometrial carcinoma (UCEC, 577 WSIs), renal cell carcinoma (RCC, 808 WSIs), and colorectal adenocarcinoma (CRC, 551 WSIs).

We conducted comprehensive external validations using 17 independent cohorts from 8 consortia and institutions (PLCO, CPTAC, YYH, CCF, SR, UHC, VU, and YU), encompassing 12 cancer types from 3 different continents. Among these external cohorts, the PLCO cohorts contain 3804 WSIs across four cancer types—bladder urothelial carcinoma (BLCA, 466 WSIs), BRCA (BRCA, 1893 WSIs), lung adenocarcinoma (LUAD, 176 WSIs), and colorectal carcinoma (CRC, 1269 WSIs)—all with disease-specific survival (DSS) status. The CPTAC cohorts contains 1871 WSIs spanning five cancer types—glioblastoma multiforme (GBM, 244 WSIs), lung squamous cell carcinoma (LUSC, 292 WSIs), pancreatic ductal adenocarcinoma (PAAD, 330 WSIs), renal cell carcinoma (RCC, 459 WSIs), and uterine corpus endometrial carcinoma (UCEC, 546 WSIs)—all of which are annotated with OS status. The YYH cohorts contains 564 WSIs comprising stomach adenocarcinoma (STAD, 265 WSIs with OS labels) and BRCA (299 WSIs with disease-free interval (DFI) status). The CCF cohort contains 322 WSIs of head and neck squamous cell carcinoma (HNSC, with OS time). The SR cohort contains 423 WSIs of CRC with DSS status annotations. The UHC cohorts comprises 158 WSIs, including 62 WSIs of lung adenocarcinoma (LUAD) and 96 WSIs of small cell lung cancer (SCLC), in which SCLC is a histological subtype absent from our training data, and during inference, it is considered lung adenocarcinoma. Despite their differences as distinct histological subtypes, LUAD was selected because of the lack of SCLC training data, serving as a substitute input for the model. The VU cohort contains 137 WSIs of HNSC with OS times. The YU cohort contains 95 WSIs of LUAD annotated with OS status.

### PROGPATH framework

We present PROGPATH, a unified pancancer prognosis prediction model that integrates histopathological image features with routinely collected clinical variables, such as age, sex, and tumor stage. PROGPATH is constructed via a weakly supervised deep learning method with auxiliary clinical features. Leveraging a foundation model for histopathological image encoding,^42,43^ PROGPATH can extract representative tile-level morphology features, which are aggregated via an attention-based multiple instance learning (AMIL) mechanism to generate whole-slide prognostic representations. To enhance prognosis prediction, PROGPATH fuses histopathological and clinical features via a cross-attention transformer model, and further employs a dynamic router-based classification strategy that optimally handles adaptive feature representations, effectively addressing pancancer heterogeneity through classifier weighting.

#### Image processing and representation learning

To extract meaningful features from WSIs, we first applied Otsu’s segmentation method^56^ to eliminate background and nontissue regions. Each WSI was then divided into nonoverlapping patches of 224×224 pixels at 10× magnification. For the encoding of these patches into meaningful representations, we employed a Vision-Transformer-based feature extractor Virchow2,^42,43^, which converts each patch into a 2560-dimensional feature vector. Subsequently, patches within a WSI were concatenated to form a comprehensive *N* × 2560 feature matrix for each slide. Here, *N* denotes the total number of patches per WSI and varies across different WSIs. Through this image processing process, we can effectively transform the raw WSIs into compact and informative features that can be utilized for survival analysis.

#### Attention multiple instance learning

To aggregate patch-level features into a slide-level representation, we adopted an AMIL approach,^44^ generating a summary embedding ${H}_{1}\in {\text{R}}^{1\times 768}$. We adopt the standard AMIL framework as the backbone for aggregating patch-level features and extend it to support multisource fusion within our unified pancancer survival architecture. The detailed AMIL implementation is provided in the Supplementary Materials.

#### Transformer-based cross-attention fusion

To bridge the semantic gap between images and clinical modalities, we introduce a transformer-style cross-attention fusion mechanism.^57^ Unlike traditional methods that directly concatenate features from different modalities, our approach explicitly learns mutual dependencies between histopathology $\left({H}_{1}\in {R}^{1\times 768}\right)$ and clinical features $\left(C\in {R}^{1\times M}\right)$. Two symmetric MLPs project them into latent space, yielding embeddings ${E}_{\text{h}}\in {\text{R}}^{1\times d}$ and ${E}_{c}\in {\text{R}}^{1\times d}$ respectively. Cross-modal attention is computed to extract clinically-relevant histopathological features ${A}_{c\to \text{h}}$ and vice versa $\left({A}_{h\to c}\right)$. This design enhances modality alignment and ensures clinically contextualized image features, which are critical for robust survival prediction.

#### Cancer-aware router for pancancer survival modeling

Unlike conventional approaches, which require training separate models for each cancer type, *n* independent networks for *n* cancer cohorts exist. Our framework, in contrast, establishes the first unified pancancer modeling paradigm capable of processing different cancer cohorts. Specifically, our framework correlates pancancer general prognostic patterns with cancer-specific knowledge through a gated routing module. To this end, we designed a cancer-aware router, inspired by the mixture of experts (MoE).^58,59^ The cancer-aware router has three key components.

- Conditional cancer control tokens. Categorical cancer-type information is encoded into a one-hot vector and transformed into a latent token via a lightweight two-layer neural network with SiLU activation and instance normalization. This token modulates downstream routing and feature fusion.
- Cancer indicator projection. The conditional cancer tokens are utilized in two ways. First, these tokens are fused with histopathological and clinical features to form the ultimate dynamic feature via channelwise concatenation. Moreover, they are employed to generate the gating weights for the dynamic router via linear projection.
- Dynamic feature routing. Instead of training *n* independent models, we route the fused representation through a shared expert pool, each tuned for a specific cancer cohort. Gating is adjusted with a logarithmic boost for the current cancer type, ensuring specificity while enabling parameter sharing. The final prediction is given by a weighted sum of the top-activated expert outputs. The detailed implementation of the cancer-aware router is provided in the Supplementary Materials.

#### Survival estimation via joint loss optimization

Survival prediction is a regression task that relies on time-to-event data consisting of time-dependent event status and its relevant observed time. Outcomes of the event may be observed or right-censored from the last follow-up. The model is trained to minimize a composite loss combining two objectives: (1) the Cox partial likelihood *L*_*cox*_, which models absolute risk, and (2) a ranking loss *L*_*rank*_,^22^ which preserves relative survival ordering among patients. The final loss is $L={L}_{{cox}}+{L}_{{rank}}$. This dual-loss strategy enhances both calibration and discrimination in survival estimates. To accommodate variable-length WSI inputs across cancer types, we implement batch-level prediction and gradient accumulation, ensuring balanced optimization across all cohorts.

#### Interpretable visualization

To reveal relatively important regions of a certain WSI that contribute to patient-level survival outcomes, we first compute forward passes for 224 × 224 patches via the AMIL module and then collect attention scores resulting from the AMIL module for each patch. Attention scores were scaled to the range of 0.0–1.0 via percentile-based normalization, where 0.0 and 1.0 correspond to the minimum and maximum percentile values of the attention distribution, representing low and high attention intensity, respectively. The scores were then interpolated to the original resolution and spatially aligned to the relevant WSI, resulting in an attention heatmap for each WSI.^60^ For visualization, the heatmap is overlaid onto the corresponding WSI with an opacity value of 0.3.

### Evaluation for prognosis prediction

We assessed the prognostic outcome for survival events associated with PROGPATH in terms of the concordance index (C-index), Kaplan–Meier curves, the log-rank significance test, and the time-dependent area under the curve (AUC). Specifically, the threshold for dividing patients into high-risk and low-risk groups was set to the median predicted risk score. The time-dependent AUC is computed by the mean value of several evenly spaced values between the 20th and 81st percentile time points.^30^ To evaluate the significance of differences between the low-risk and high-risk groups in the Kaplan–Meier analysis, we performed a two-sided log-rank test, and the results were considered significant if *p* < 0.05. For the TCGA held-out validation, we aggregated the predictions from 5-fold cross-validation (stratified by cancer type) to obtain the overall C-index, AUC, and Kaplan–Meier curve results. For the external validation sets, we used the full dataset to perform model inference. For the held-out test sets, we used the Mann–Whitney U test to compare the overall performance of the methods. For the external validation datasets, we performed a two-sided z test for each dataset. Statistical significance was defined as *p* < 0.05 for all comparisons.

### Comparison details of the baseline methods

We conducted fair comparisons for all the comparison baselines, including: (1) MCAT (https://github.com/mahmoodlab/MCAT), (2) MOTCAT (https://github.com/Innse/MOTCat), (3) SurvPath (https://github.com/mahmoodlab/SurvPath), and (4) Cox proportional hazards models implemented in Lifelines.^61^ For the compared multimodal methods, we adapt their architectures to enable the same input used in PROGPATH. We replaced the genomic inputs with clinical variables (age, sex, and tumor stage) while preserving their original network structures. Specifically, for all the compared multimodal methods, we used the Virchow2 foundation model to extract patch-level histopathological features, ensuring consistency in image processing across methods. For the Cox baseline model, we implemented a Cox proportional hazards model using only the clinical variables. Regression coefficients were estimated on TCGA training cohorts via partial likelihood, and the resulting model was directly applied to external datasets to generate risk predictions. All the models were trained and evaluated via the same data splits, input features, and training protocols to ensure consistent and reproducible comparisons.

### Implementation details

In this study, all patients from the TCGA cohorts were pooled together and randomly split into nonoverlapping training and validation sets (4:1) in a 5-fold cross-validation manner, stratified by cancer type. The remaining 17 cohorts from PLCO, CPTAC, YYH, CCF, VU, SR, YU, and UHC are used as externally independent validations. We adopted AdamW^62^ as the optimizer with a learning rate of 1e-4 and cosine annealing weight decay. The training procedure is set to 20 epochs.^29,31^ Owing to the varying dimensions of WSI patches, we implemented a batch size of 256 via explicit stacking and aggregating the predictions and gradients to maintain balanced representation of all cancer types within each batch. The model was developed with the PyTorch deep learning framework and runs on an NVIDIA RTX A6000 GPU.

## Supplementary information

SUPPLEMENTAL MATERIAL

## Data Availability

The TCGA cohorts histopathology and clinical data used in this study are publicly available at the cBioPortal (https://www.cbioportal.org/) and the National Cancer Institute Genomic Data Commons Portal (https://gdc.cancer.gov/about-data/publications/pancanatlas). The CPTAC WSIs and clinical data are publicly available at The Cancer Imaging Archive (https://proteomics.cancer.gov/programs/cptac). The PLCO data are available at https://cdas.cancer.gov/plco/, and the SR data are available at 10.6019/S-BIAD1285).

## Code availability

Our work is publicly available at https://github.com/Valeyards/ProgPath, and includes installation, model weights, and example data.
