# Supplementary material for: Pancancer outcome prediction via a unified weakly supervised deep learning model
Source: Signal Transduct Target Ther. 2025 Sep 3;10:285. doi: 10.1038/s41392-025-02374-w (PMC12405520; doi:10.1038/s41392-025-02374-w)
Supplement: Supplementary file 1 — SUPPLEMENTAL MATERIAL [file 41392_2025_2374_MOESM1_ESM.docx]

Supplementary Materials for

Pancancer outcome prediction via a unified weakly supervised deep learning model

Wei Yuan**^†^**, Yijiang Chen**^†^**, Biyue Zhu, Sen Yang, Jiayu Zhang, Ning Mao, Jinxi Xiang, Yuchen Li, Yuanfeng Ji, Xiangde Luo, Kangning Zhang, Xiaohan Xing, Shuo Kang, Dongyuan Xiao, Fang Wang, Jinkun Wu, Haiyan Zhang, Hongping Tang, Himanshu Maurya, German Corredor, Cristian Barrera, Yufei Zhou, Krunal Pandav, Junhan Zhao, Prantesh Jain, Luke Delasos, Junzhou Huang, Kailin Yang, Theodoros N Teknos, James Lewis Jr, Shlomo Koyfman, Nathan A. Pennell, Kun-Hsing Yu, Xiao Han, Jing Zhang*, Xiyue Wang*, Anant Madabhushi

**†**Contributed equally (co-first)

*Correspondence to Xiyue Wang(xiyuew@stanford.edu) and Jing Zhang (jing_zhang@scu.edu.cn)

**This PDF file includes:**

Materials and Methods

Figures S1 to S11

Tables S1 to S8

**Materials and Methods**

**Methods**

Attention multiple instance learning

In multiple-instance learning1,2, bag (whole-slide image, WSI) level prediction is made through aggregating instances (patch information) supervised by a slide-level signal (i.e., survival status and survival time). Following the image processing pipeline, information within each WSI is compressed into one $\text{N}\text{×2560}$ vector. The attention-based multiple instance learning (AMIL) includes an attention pooling module $\text{ℱ}_{\text{a}}$ and a Multilayer Perceptron (MLP) projection layer $\text{P}_{\text{1}}\text{∈}\text{ℝ}^{\text{768×2560}}$. For an input bag embedding $\text{H}\text{∈}\text{ℝ}^{\text{N}\text{×2}\text{560}}$, the slide representation $\text{H}_{\text{1}}$ given by AMIL module can be written as:

$$\begin{aligned} \text{H}_{\text{1}}\text{ }\text{=}{\text{ }\text{ℱ}}_{\text{a}}\left( \text{P}_{\text{1}}\left( \text{H} \right) \right)\#\left( \text{1} \right) \end{aligned}$$

First, $\text{H}$ is passed to $\text{ℱ}_{\text{a}}$, in which the aggregation for feature embeddings is computed by:

$$\begin{aligned} \text{z}\text{ }\text{=}\text{ }\sum_{\text{n}\text{=1}}^{\text{N}} \text{S}_{\text{n}}\text{ℎ}_{\text{n}}\#\left( \text{2} \right) \end{aligned}$$

where $\text{ℎ}_{\text{n}}$ is the feature embedding for one patch $\text{ℎ∈}\text{ℝ}^{\text{1×7}\text{68}}$, and the attention score $\text{S}$ is computed by:

$$\begin{aligned} \text{S}_{\text{n}}\text{ }\text{=}\text{ }\frac{\exp\left( \text{W}\left( \tanh\left( \text{U}\text{ℎ}_{\text{n}}^{\text{T}} \right) \right)\text{⊙sigm}\left( \text{V}\text{ℎ}_{\text{n}}^{\text{T}} \right) \right)}{\sum_{\text{n}\text{=1}}^{\text{N}} \exp\left( \text{W}\left( \tanh\left( \text{U}\text{ℎ}_{\text{n}}^{\text{T}} \right) \right)\text{⊙sigm}\left( \text{V}\text{ℎ}_{\text{n}}^{\text{T}} \right) \right)}\#\left( \text{3} \right) \end{aligned}$$

where$\text{W}\text{∈}\text{ℝ}^{\text{1×3}\text{84}}\text{,}\text{ }\text{U}\text{∈}\text{ℝ}^{\text{1×7}\text{68}}\text{,}\text{ }\text{V}\text{∈}\text{ℝ}^{\text{3}\text{84}\text{×7}\text{68}}$denote projection weights, $\text{⊙}\text{ }$denotes element-wise multiplication. The symbols $\text{tan}\text{ℎ}$ and $\text{sigm}$ represent the tanh and sigmoid activation functions. During evaluation, slides within each patient are aggregated to form a patient-level prediction by maxpooling the predicted prognostic risk score of all slides.

Cancer-aware router

To enable unified, pan-cancer survival modeling while maintaining discriminative disease-specific representations, our framework correlates pan-cancer general prognostic patterns with cancer-specific knowledge through a gated routing module. The cancer-aware router has three key components:

• Conditional cancer control tokens. The input cancer type information is first encoded as an $\text{n}\text{−}\text{d}\text{imensional}$ one-hot vector as:

$$\begin{aligned} \text{C}_{\text{ik}}\text{ }\text{=}\text{ }\left\{ \begin{matrix} \text{0,} & \text{if }\text{k}\text{≠}\text{i} \\ \text{1,} & \text{otherwise} \end{matrix} \right.\text{ }\text{k}\text{=1,2,...,}\text{n}\#\left( \text{4} \right) \end{aligned}$$

where n denotes the total number of cancer types, $\text{C}_{\text{ik}}\text{=}\text{1}$ signifies that the prognostic task on k-th cancer type is currently being executed. This one-hot feature is then encoded by a two layer neural network to form cancer tokens $\text{F}_{\text{ca}}\text{∈}\text{ℝ}^{\text{d}_{\text{1}}}$, formulated as:

$$\begin{aligned} \text{F}_{\text{ca}}\text{ }\text{=}\text{ }\text{σ}\left( \text{W}_{\text{2}}\text{N}\left( \text{σ}\left( \text{W}_{\text{1}}\text{x}\text{+}\text{b}_{\text{1}} \right) \right)\text{+}\text{b}_{\text{2}} \right)\#\left( \text{5} \right) \end{aligned}$$

where $\text{σ}$ denotes SiLU activation function, $\text{N}$ denotes instance normalization, and $\{\text{W}_{\text{1}}\text{,}\text{W}_{\text{2}}\text{,}\text{b}_{\text{1}}\text{,}\text{B}_{\text{2}}\}$ denotes the parameters of fully-connected layers.

• Cancer indicator projection. The conditional cancer tokens are utilized by 1) representing the ultimate dynamic feature $\text{Z}$ by channel-wise concatenation of feature representations $\text{Z}\text{=}\left[ \text{A}_{\text{ℎ→}\text{c}}\text{,}\text{A}_{\text{c}\text{→ℎ}}\text{,}\text{ℱ}_{\text{ca}} \right]\text{∈}\text{ℝ}^{\text{2}\text{d}\text{+}\text{d}_{\text{1}}}$. 2) generating gating weights $\text{G}_{\text{ca}}\text{∈}\text{ℝ}^{\text{n}}$ for the dynamic router via projection of cancer tokens:

$$\begin{aligned} \text{G}_{\text{ca}}\text{ }\text{=}\text{ }\text{Softmax}\left( \frac{\text{W}_{\text{g}}\text{F}_{\text{ca}}\text{+}\text{b}_{\text{g}}\text{+}\text{M}_{\text{b}}}{\text{τ}} \right)\#\left( \text{6} \right) \end{aligned}$$

where $\text{W}_{\text{g}}$ is projection transformation, $\text{b}_{\text{g}}$ is learnable bias, $\text{τ}$ is the temperature coefficient, and $\text{M}_{\text{b}}$ is a positional bias that highlights the current cancer type.

• Dynamic feature Router. Instead of requiring multiple specialized or fixed models for different prognostic tasks, our dynamic feature router estimates patient risks from different cancer types in a unified manner. Modulated by $\text{G}_{\text{ca}}$, the dynamic router is able to route $\text{Z}$ through a set of parallel prognostic heads $\{\text{E}_{\text{i}}{\}}_{\text{i}\text{=1}}^{\text{n}}$, each specialized for a particular cancer type. The final estimation of patient risk is calculated as:

$$\begin{aligned} \hat{\text{y}}\text{ }\text{=}\text{ }\sum_{\text{i}\text{=1}}^{\text{n}} \text{ }{\hat{\text{G}}}_{\text{ca}}^{\left( \text{i} \right)}\text{E}_{\text{i}}\left( \text{Z} \right)\#\left( \text{7} \right) \end{aligned}$$

where $\text{E}_{\text{i}}\left( \text{⋅} \right)$ is i-th cancer-specific prognostic head, ${\hat{\text{G}}}_{\text{ca}}$ is the final adaptive weights of $\{\text{E}_{\text{i}}{\}}_{\text{i}\text{=1}}^{\text{n}}$, computed by:

$$\begin{aligned} {\hat{\text{G}}}_{\text{ca}}\text{ }\text{=}\text{ }\text{Softmax}\left( \text{G}_{\text{ca}}^{\text{to}\text{p}} \right)\#\left( \text{8} \right) \end{aligned}$$

and $\text{G}_{\text{ca}}^{\text{top}}$ is computed by retaining the top-k entries of $\text{G}_{\text{ca}}$, and a logarithmic boost $\ln\left( \text{β} \right)$ to the current cancer type $\text{i}$:

$$\begin{aligned} \text{G}_{\text{ca}}^{\text{top}}\left[ \text{j} \right]\text{ }\text{=}\text{ }\left\{ \begin{matrix} \text{G}_{\text{ca}}\left[ \text{j} \right]\text{+}\ln\left( \text{β} \right)\text{,} & \text{j}\text{ }\text{∈}\text{ }\text{top-}\text{k}\text{ and }\text{j}\text{=}\text{i} \\ \text{G}_{\text{ca}}\left[ \text{j} \right]\text{,} & \text{j}\text{ }\text{∈}\text{ }\text{top-}\text{k}\text{ and }\text{j}\text{≠}\text{i} \\ \text{−∞,} & \text{j}\text{ }\text{∉}\text{ }\text{top-}\text{k} \end{matrix} \right.\#\left( \text{9} \right) \end{aligned}$$

**Ablation Studies**

To validate the effectiveness of our newly proposed PROGPATH approach, we conducted comprehensive ablation studies to quantify the contribution of key components in the PROGPATH framework, including ablation study of the newly presented router module (the cancer-aware branch), ablation study of different feature extractors, and ablation study of feature fusion strategies. All experiments used the same hyperparameter settings and cross-validation protocols to ensure comparability.

Ablation of cancer-aware branch

First, to isolate the impact of the cancer-aware branch, we replaced the cancer-aware gated router with a standard neural classifier while maintaining other modules and factors. Results are shown in Supplementary Fig. 8 and Supplementary Table 6, that the dynamic router has a C-index performance gain of +2.4% and +4.2% in TCGA and external datasets respectively, reflecting its effectiveness.

Ablation of feature fusion strategy

To investigate the effects of feature fusion method, we compared two variants of PROGPATH: one without the Transformer cross attention, and one replaced the concatenation of features with bilinear fusion method. As shown in Supplementary Fig. 9and Supplementary Table 7, the fusion strategy used in PROGPATH led to C-index improvements of +0.6% and +3.2% in TCGA datasets, +3.7% and +2.2% in external datasets. It is noted that the newly presented Transformer-based feature fusion is more generalizable to out-of-distribution data, and the direct concatenation is more effective than bilinear fusion.

Ablation of feature extractors

Furthermore, we compared four different pathological feature extractors, including: RetCCL3, UNI4, GigaPath5, and Virchow26 in TCGA, CPTAC, and PLCO cohorts. Corresponding results are shown in Supplementary Fig. 10 and Supplementary Table 8. It turns out that Virchow2 is the most competitive feature extractor in both metrics C-index and AUC, outperforming the next-best foundation model UNI +1.7% and +1.5% in TCGA and external datasets respectively.

**Comparison of PROGPATH against Cox model**

We further assessed the prognostic performance of PROGPATH. To assess its effectiveness, we compared PROGPATH with cancer-specific cox-proportional hazards models7 built on routine clinical variables—age, sex, and tumor stage. (Supplementary Fig. 1 and Supplementary Table 3).

PROGPATH consistently outperformed the clinical-data-based Cox model across all evaluations. In the TCGA held-out cohort, PROGPATH achieved an overall C-index of 0.731 and AUC of 0.737, surpassing the Cox model by 9.3% (0.669) and 8.5% (0.679), respectively (Supplementary Fig. 1a and Supplementary Table 3).

We performed extensive validation on 17 external cohorts and achieved overall C-indices of 0.727 on PLCO, 0.664 on CPTAC, and 0.677 on institutional cohorts, exceeding the Cox model by large margins (0.439 C-index on PLCO, 0.497 C-index on CPTAC, and 0.524 C-index on institutional cohorts) (Supplementary Fig. 1b, Supplementary Table 3). Similarly, PROGPATH surpassed the Cox model in AUC scores across these cohorts: 0.745 vs. 0.452 (PLCO), 0.691 vs. 0.502 (CPTAC), and 0.702 vs. 0.505 (institutional cohorts) (Supplementary Table 3). Specifically, PROGPATH demonstrated distinctive stratification performance in PLCO-BLCA and PLCO-BRCA (*p*<0.01), while the Cox model failed to significantly separate survival curves correctly (C-index<0.5, Supplementary Table 3). In the lung cancer cohorts, although the Cox model performed well in PLCO-LUAD, it failed to generalize to other datasets (CPTAC-LUSC, YU-LUAD, UHC- LUAD, CCF-LUAD and UHC-SCLC), with C-indices below 0.5. By contrast, PROGPATH achieved a mean C-index of 0.685 across these external datasets, underscoring its superior generalizability and robustness in multi-center validation scenarios.

**Model Interpretability**

To enhance the potential interpretability of our PROGPATH model, we conducted an in-depth analysis to identify and visually represent WSIs using heatmaps. This approach also highlights the key regions of interest (ROIs) that significantly contribute to the prognosis predictions (Supplementary Fig. 11). Through visual inspection of these attention highlights, we have made intriguing discoveries regarding the high-attention regions identified by PROGPATH in high-risk outcomes. These regions often exhibit signs of poorer cell differentiation, indicating a higher degree of cellular abnormality. Additionally, areas of necrosis, which are indicative of tissue death, are more prevalent in these high-risk regions. These findings suggest that the presence of these histopathology characteristics may be associated with a more aggressive tumor phenotype and a worse prognosis. Conversely, in low-risk slides, PROGPATH tends to focus its attention on histopathology regions characterized by well-differentiated cells with uniformly arranged nuclei. Tumor cells are sparse, arranged in papillary, glandular, or cord-like patterns, set against a backdrop of abundant stroma marked by hyaline degeneration, edema, and mucinous changes. This stroma is consistently infiltrated by chronic inflammatory cells, such as lymphocytes and plasma cells. Red blood cell extravasation and hemosiderin deposition are frequently observed, and neoplastic blood vessels are rare. We used three cancer types, namely UCEC, RCC, and BRCA as examples. For each cancer type, we depicted the areas within WSIs that received heightened attention in both high-risk and low-risk groups.

WSIs associated with UCEC showed a notable distinction between tumors predicted as high-risk survival outcomes (Supplementary Fig. 11a) and those predicted as low-risk (Supplementary Fig. 11b). High-risk tumors in these images are characterized by poor differentiation, featuring diffuse solid cell growth and an abundance of cells. Moreover, these tumors display more aggressive micropapillary structures (Supplementary Fig. 11a). In contrast to high-risk areas, the low-risk region shows tumor cells arranged in dilated glandular ducts, often accompanied by apoptosis and scattered degeneration. The lumen frequently contains eosinophilic or basophilic secretions. The stroma is abundant, loose, and edematous, with areas of collagen degeneration. It is infiltrated by lymphocytes and neutrophils. Inflammatory necrosis and abscess formation are less frequent (Supplementary Fig. 11b).

WSIs related to RCC also showed differences between the high-risk and low-risk survival groups. In these high-risk areas (Supplementary Fig. 11c), there is a significantly increased cell density, accompanied by an obvious elevation in the nuclear-to-cytoplasmic ratio. Additionally, more graphic necrosis and fatty infiltration are also observed. However, in low-risk regions (Supplementary Fig. 11d), tumor cells are sparse, often forming papillary structures and dilated glandular ducts. The lumen of these ducts frequently contains eosinophilic or basophilic secretions. Tumor cells exhibit clear and abundant cytoplasm with scattered nuclear degeneration. The stroma is loose, edematous, and rich, with significant red blood cell extravasation and hemosiderin deposition. Scattered lymphocytes, plasma cells, and neutrophils infiltrate the stroma, reflecting a less aggressive inflammatory response compared to high-risk areas.

The examination of WSIs related to BRCA revealed distinctive features between high-risk and low-risk slides (Supplementary Fig. 11e and Supplementary Fig. 11f). In the high-risk image (Supplementary Fig. 11e), high-attention patches showed higher nuclear density, diffuse distribution, pronounced nuclear polymorphism, marked chemotaxis, and markedly wide and narrow nuclear spacing. Conversely, in the low- risk image (Supplementary Fig. 11f), tumor cells are sparse and arranged in parallel cord-like patterns, with abundant amphophilic cytoplasm often containing intracytoplasmic inclusions. The surrounding stroma is rich, displaying hyaline degeneration and mucinous changes. It is infiltrated by lymphocytes, plasma cells, and occasional eosinophils, reflecting a subdued inflammatory response. Neoplastic blood vessels are minimal, consistent with the low-risk prognosis.

**Figure. S1.**


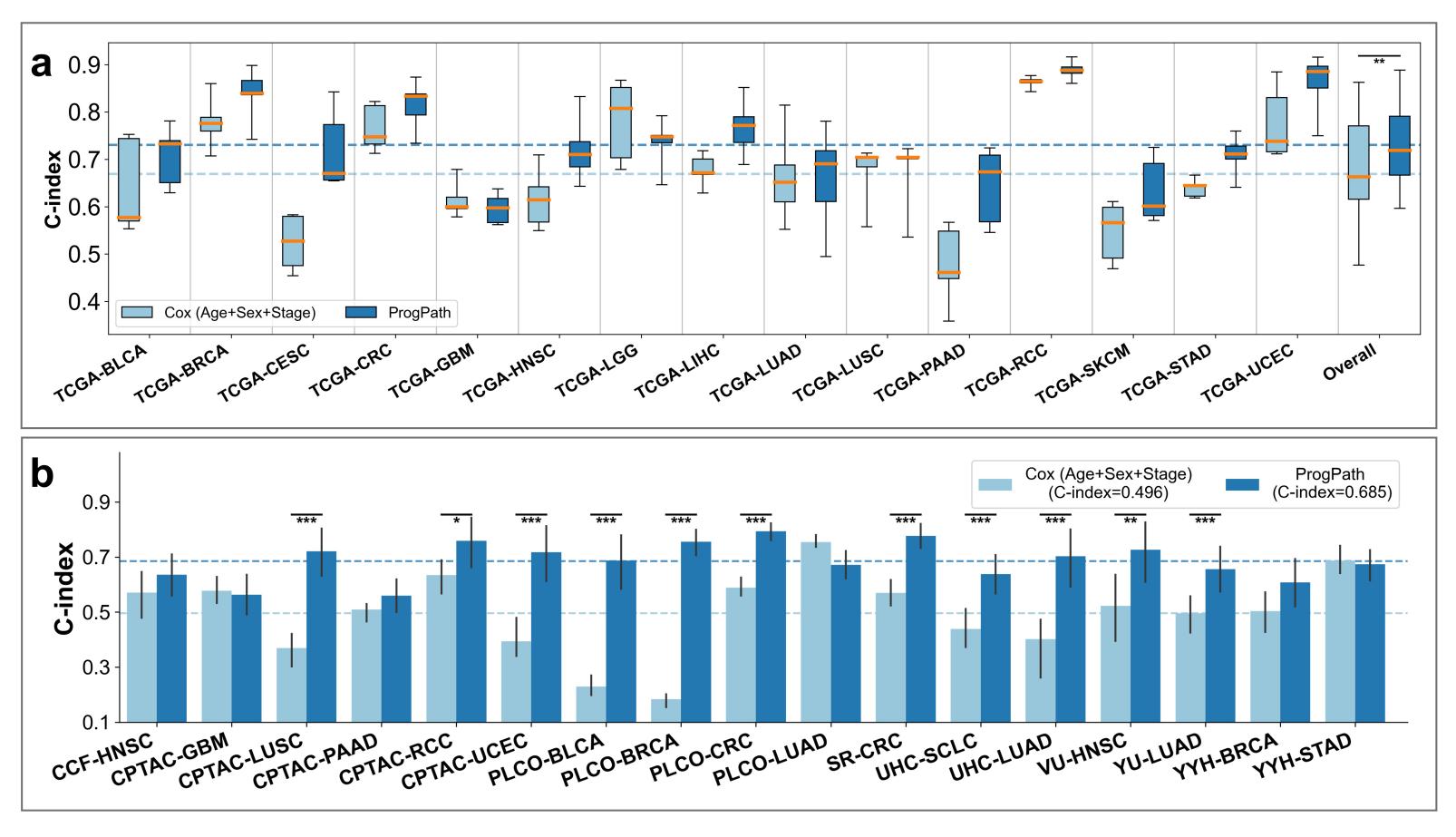


**Supplementary Figure 1: Prognostic performance of PROGPATH against a Cox model.** We show C-index comparisons in the TCGA cohort and three types of external validation cohorts: PLCO, CPTAC, and independent clinical cohorts. **a** Box plots comparing the C-index of PROGPATH and a standard Cox proportional hazards model across the 15 TCGA cancer types. The Cox model takes clinical variables (age, sex, and stage) as the inputs. PROGPATH consistently achieves higher overall performance (*p*<0.01). The overall box plots summarize the distribution of model performance (mean and standard deviation) across cancer types, with statistical significance indicated. P values were calculated via the Mann–Whitney U test on the basis of the mean 5-fold performance of each method. **b** Bar plots comparing the C-index of PROGPATH and a standard Cox proportional hazards model across external cohorts with significant markers. PROGPATH significantly outperforms the Cox model across 11 out of 17 datasets. The error bars represent 95% confidence intervals estimated via the bootstrap method with 1000 replicates. P values were calculated via a two-sided z test on the basis of the bootstrap samples. In **a** and **b**, dashed horizontal lines reflect the average C-index per model. Significant markers are defined as follows: * for *p*<0.05, ** for *p*<0.01, and *** for *p*<0.001.

**Figure. S2.**


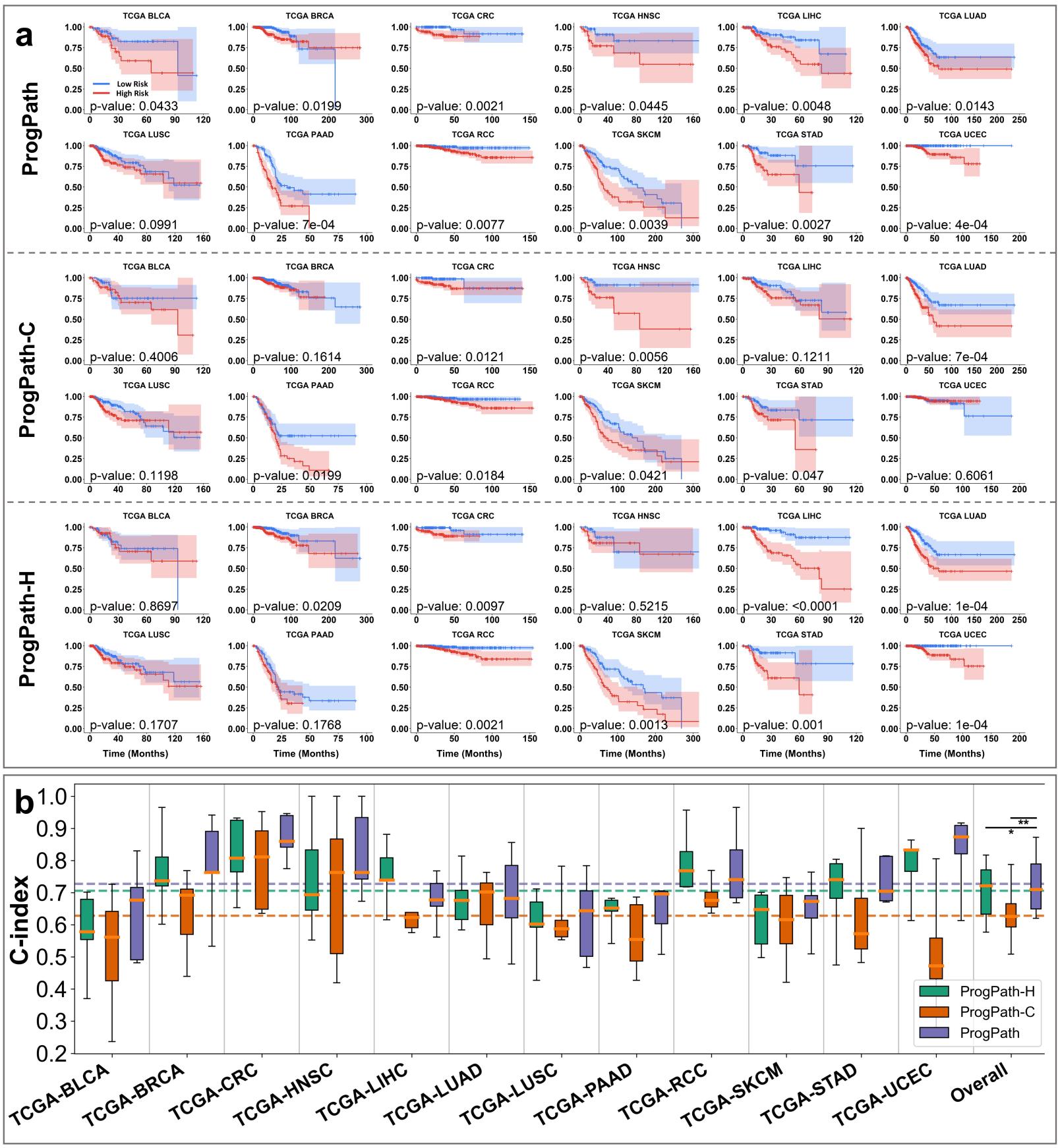


**Supplementary Figure 2: PROGPATH predicts the prognosis of early-stage (stage I-II) cancer patients by comparing WSI-only (PROGPATH-H) and clinical-only (PROGPATH-C) approaches in TCGA held-out datasets. a** Kaplan‒Meier analysis across TCGA held-out cohorts. All Kaplan‒Meier curves are plotted with 95% confidence intervals (CIs). Overall, PROGPATH successfully stratified patients into different risk groups (log-rank test *p*<0.05) in 11 out of 12 TCGA cancer types. PROGPATH-H achieved significant stratification in 8 out of 12 cancer types, and PROGPATH-C achieved significant stratification in 7 out of 12 cancer types. **b** C-index comparison between PROGPATH-H, PROGPATH-C, and PROGPATH.

**Figure. S3.**


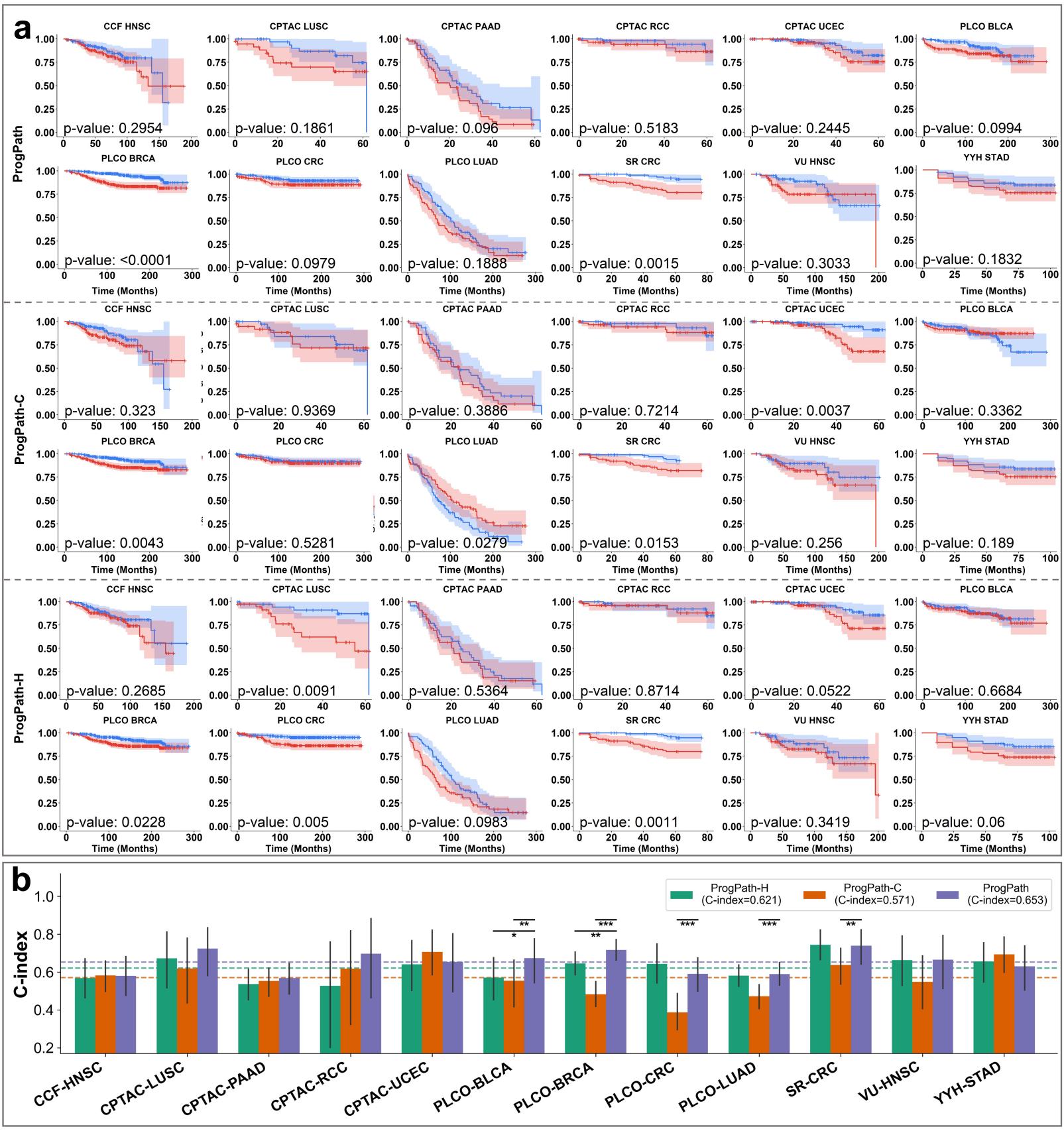


**Supplementary Figure 3: PROGPATH predicts the prognosis of early-stage (stage I-II) cancer patients by comparing WSI-only (PROGPATH-H) and clinical-only (PROGPATH-C) approaches in external datasets. a** Kaplan‒Meier analysis across external cohorts, including PLCO, CPTAC and in- dependent clinical cohorts. All Kaplan‒Meier curves are plotted with 95% confidence intervals (CIs). **b** Comparison of C-index values among PROGPATH-H, PROGPATH-C, and PROGPATH across external datasets, presented as bar plots. Overall, PROGPATH has C-index performance gain of +4.5% and +13.1% compared to PROGPATH-H and PROGPATH-C, respectively.

**Figure. S4.**


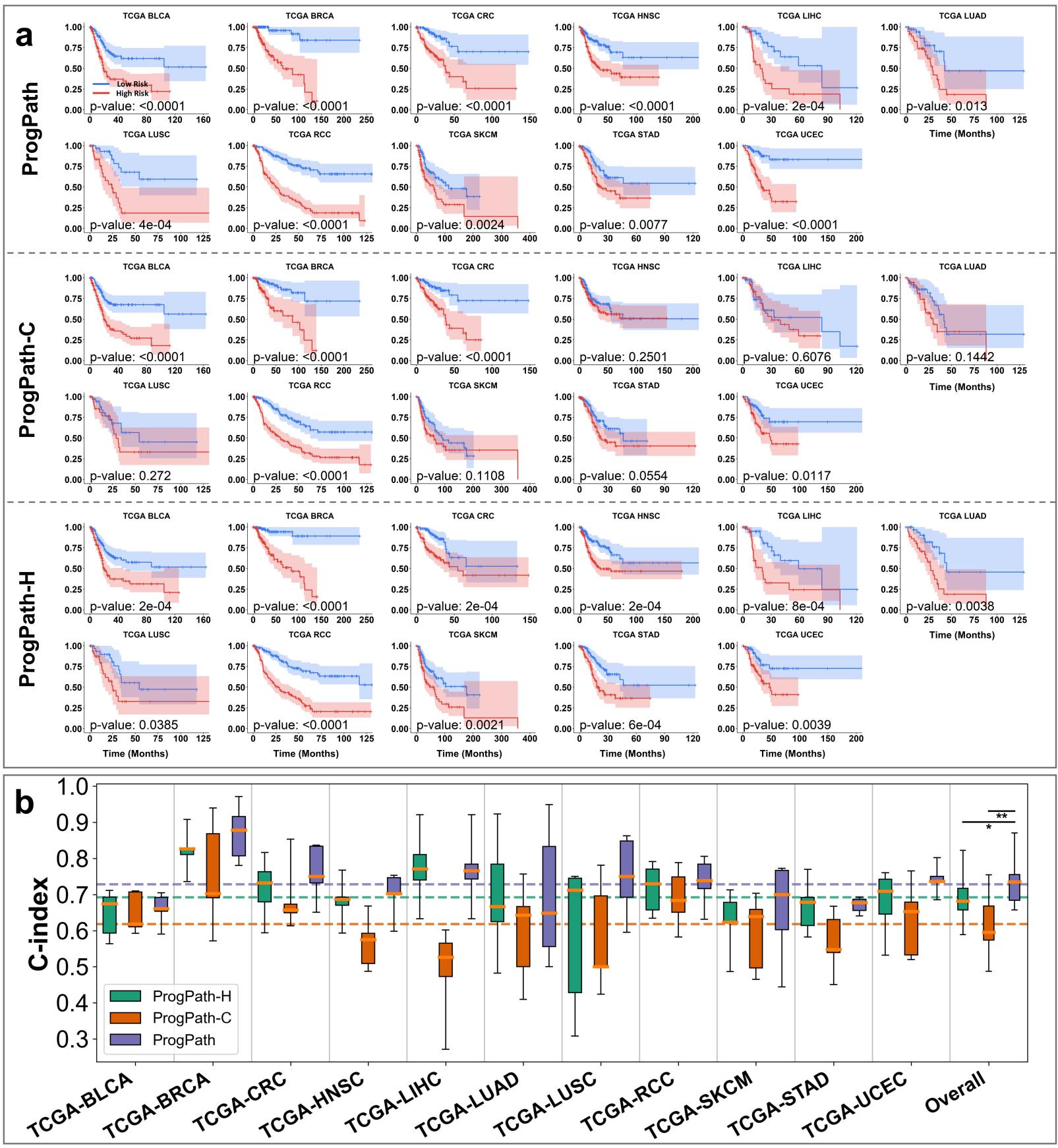


**Supplementary Figure 4: PROGPATH can predict the prognosis of late-stage (stage III-IV) cancer patients by comparing WSI-only (PROGPATH-H) and clinical-only (PROGPATH-C) approaches in TCGA held- out datasets. a** Kaplan‒Meier analysis in the TCGA held-out cohorts. All Kaplan‒Meier curves are plotted with 95% confidence intervals (CIs). Overall, PROGPATH and PROGPATH-H successfully stratified patients into different risk groups (log-rank test *p*<0.05) in all 11 TCGA cancer types. PROGPATH-C successfully stratified patients into different risk groups in 5 out of 11 cancer types. **b** Comparison of C-index values among PROGPATH-H, PROGPATH-C, and PROGPATH, presented as bar plots.

**Figure. S5.**


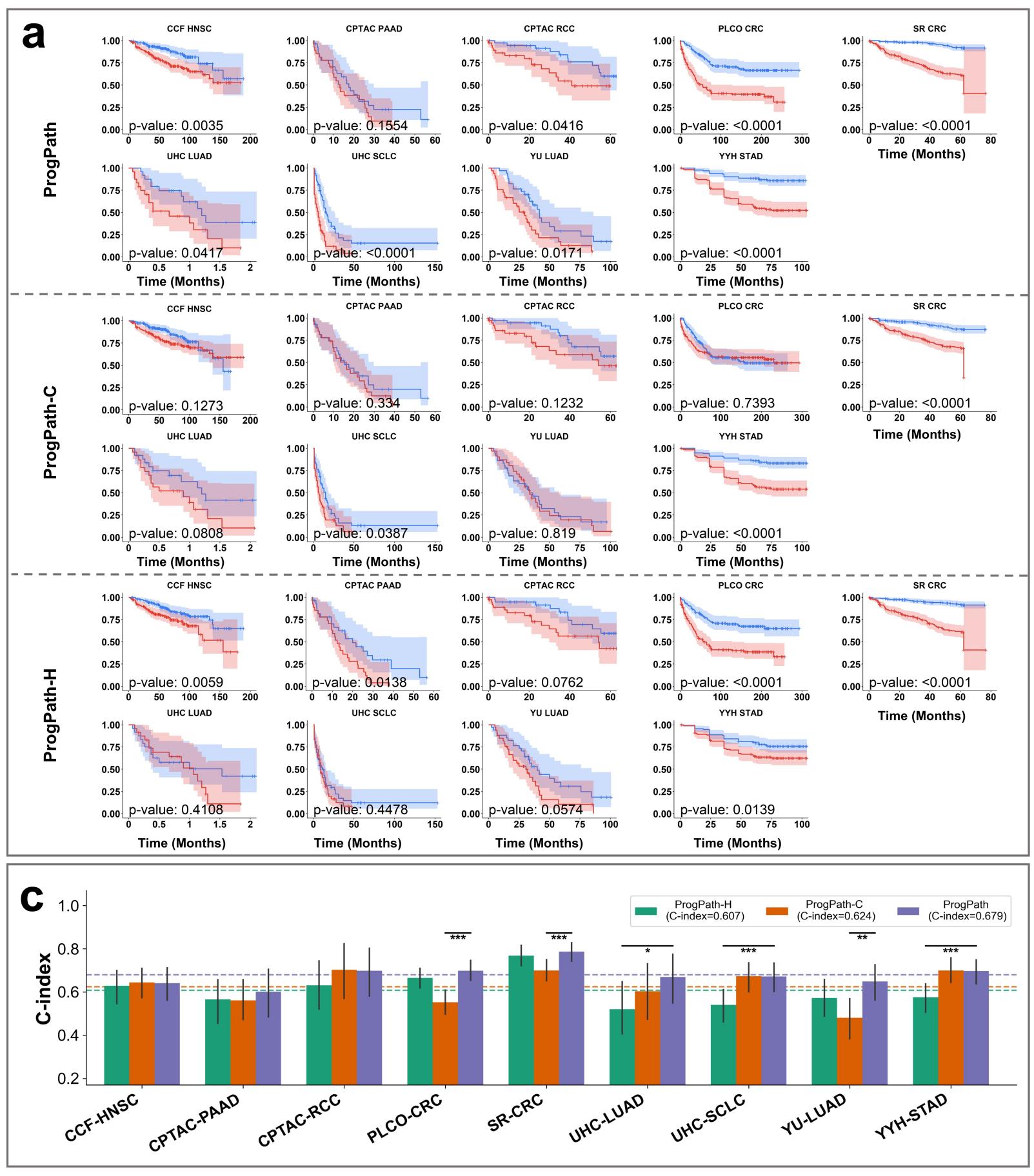


**Supplementary Figure 5: PROGPATH can predict the prognosis of late-stage (stage III-IV) cancer patients by comparing WSI-only (PROGPATH-H) and clinical-only (PROGPATH-C) approaches in external datasets. a** Kaplan‒Meier analysis across external cohorts, including PLCO, CPTAC and independent clinical cohorts. All Kaplan‒Meier curves are plotted with 95% confidence intervals (CIs). **b** Comparison of C-index values among PROGPATH-H, PROGPATH-C, and PROGPATH, presented as bar plots. Overall, PROGPATH has C-index performance gain of +11.9% and +8.8% compared to PROGPATH-H and PROGPATH-C.

**Figure. S6.**


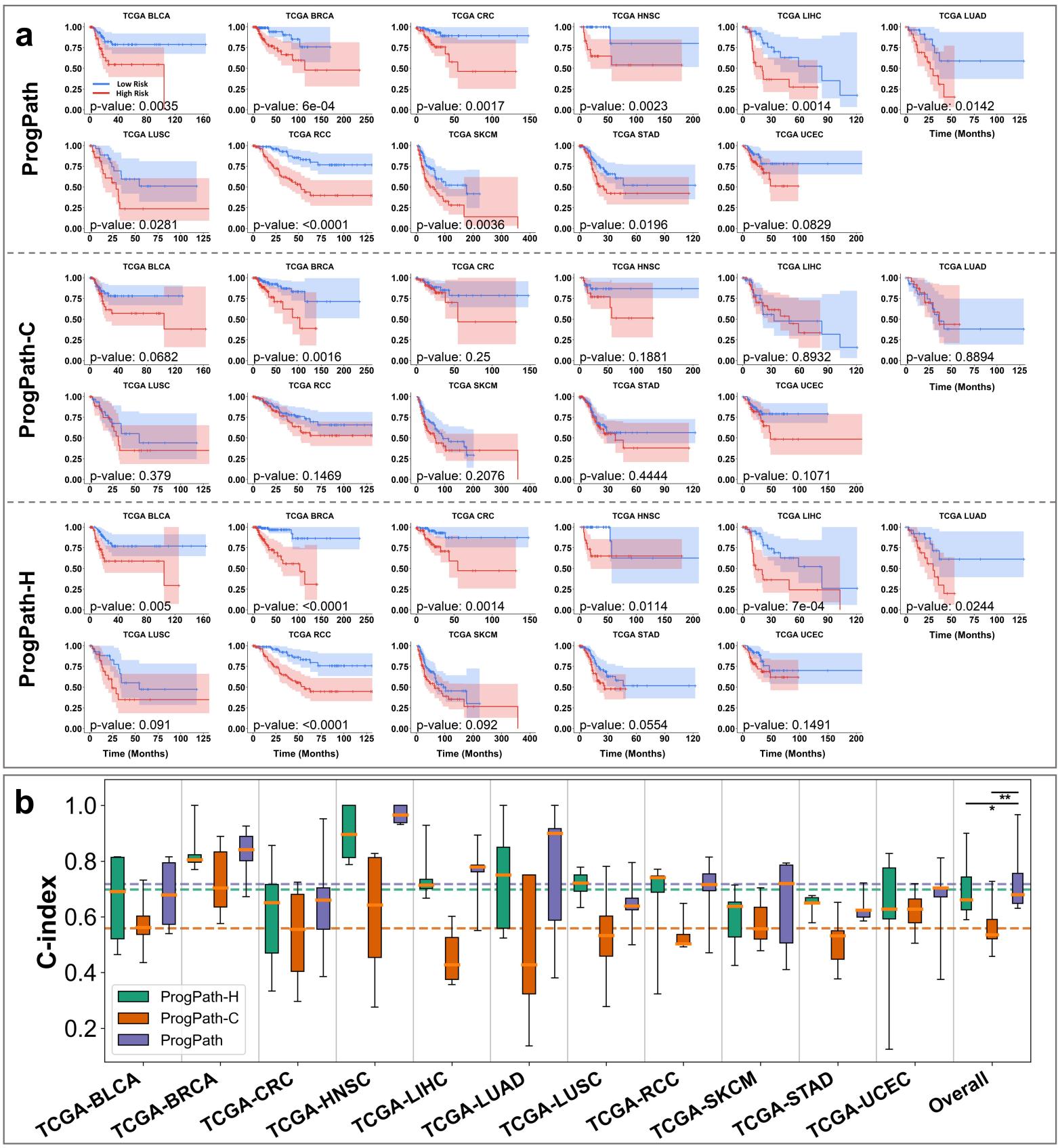


**Supplementary Figure 6: PROGPATH can predict the prognosis of stage III cancer patients by comparing WSI-only (PROGPATH-H) and clinical-only (PROGPATH-C) approaches in TCGA held-out datasets. a** Kaplan‒Meier analysis in the TCGA held-out cohorts. All Kaplan‒Meier curves are plotted with 95% confidence intervals (CIs). Overall, PROGPATH successfully stratified patients into different risk groups (log- rank test *p*<0.05) in 10 out of 11 cancer types. PROGPATH-H successfully stratified patients into different risk groups in 7 out of 11 TCGA cancer types. And PROGPATH-C successfully stratified patients into different risk groups in 1 out of 12 cancer types. **b** Comparison of C-index values among PROGPATH-H, PROGPATH-C, and PROGPATH, presented as bar plots.


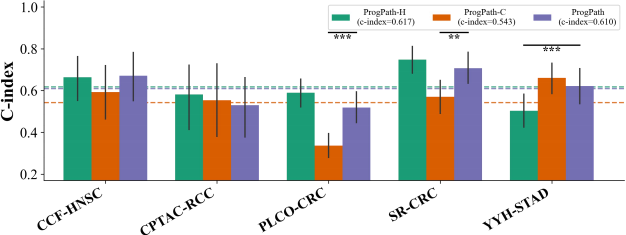


**Figure. S7.**


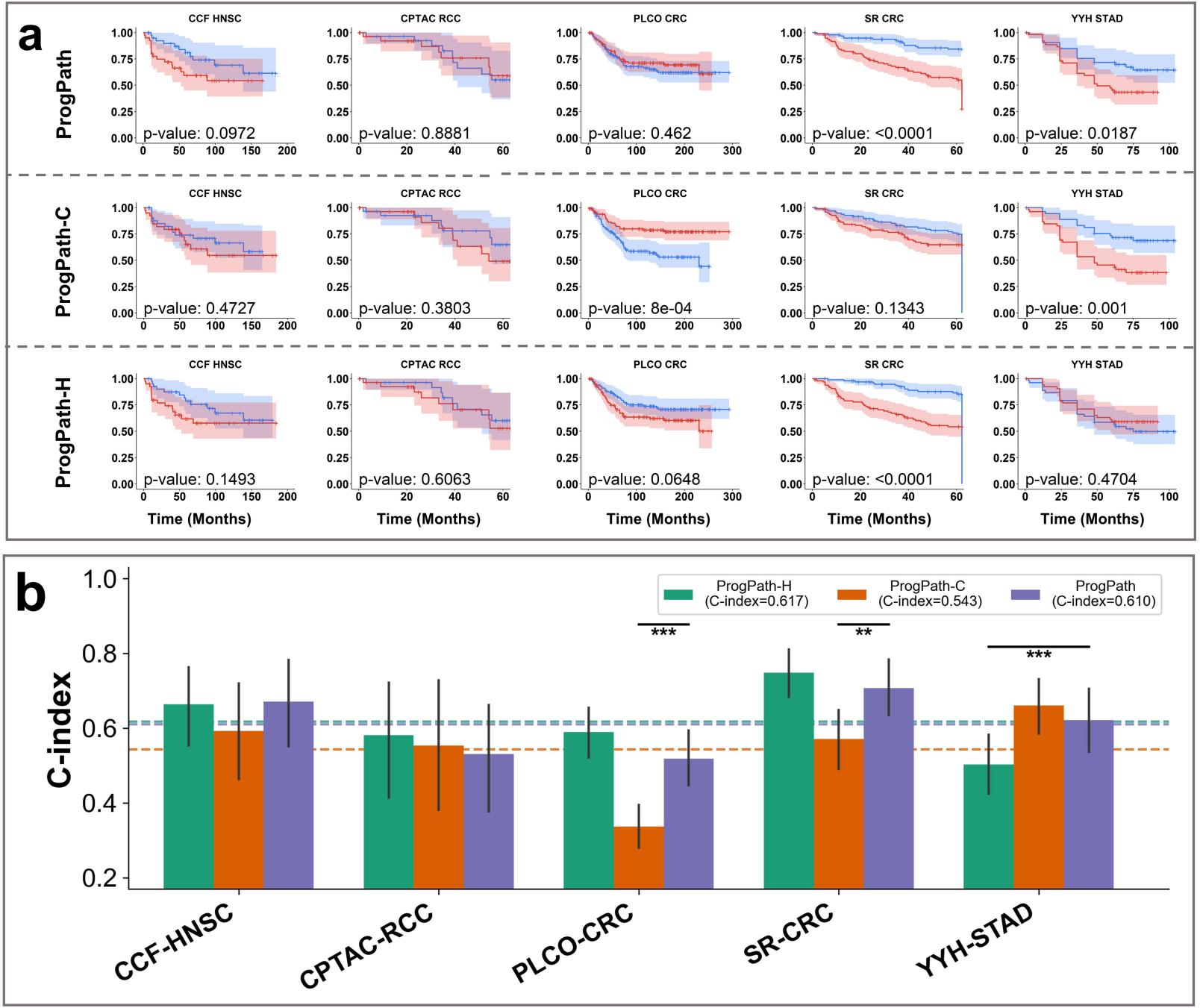


**Supplementary Figure 7: PROGPATH can predict the prognosis of stage III cancer patients by comparing WSI-only (PROGPATH-H) and clinical-only (PROGPATH-C) approaches in external datasets. a** Kaplan‒Meier analysis across external cohorts, including PLCO, CPTAC and independent clinical cohorts. All Kaplan‒Meier curves are plotted with 95% confidence intervals (CIs). **b** Comparison of C-index values among PROGPATH-H, PROGPATH-C, and PROGPATH, presented as bar plots. Overall, PROGPATH has C-index performance gain of +12.3% compared to PROGPATH-C.

**Figure. S8.**


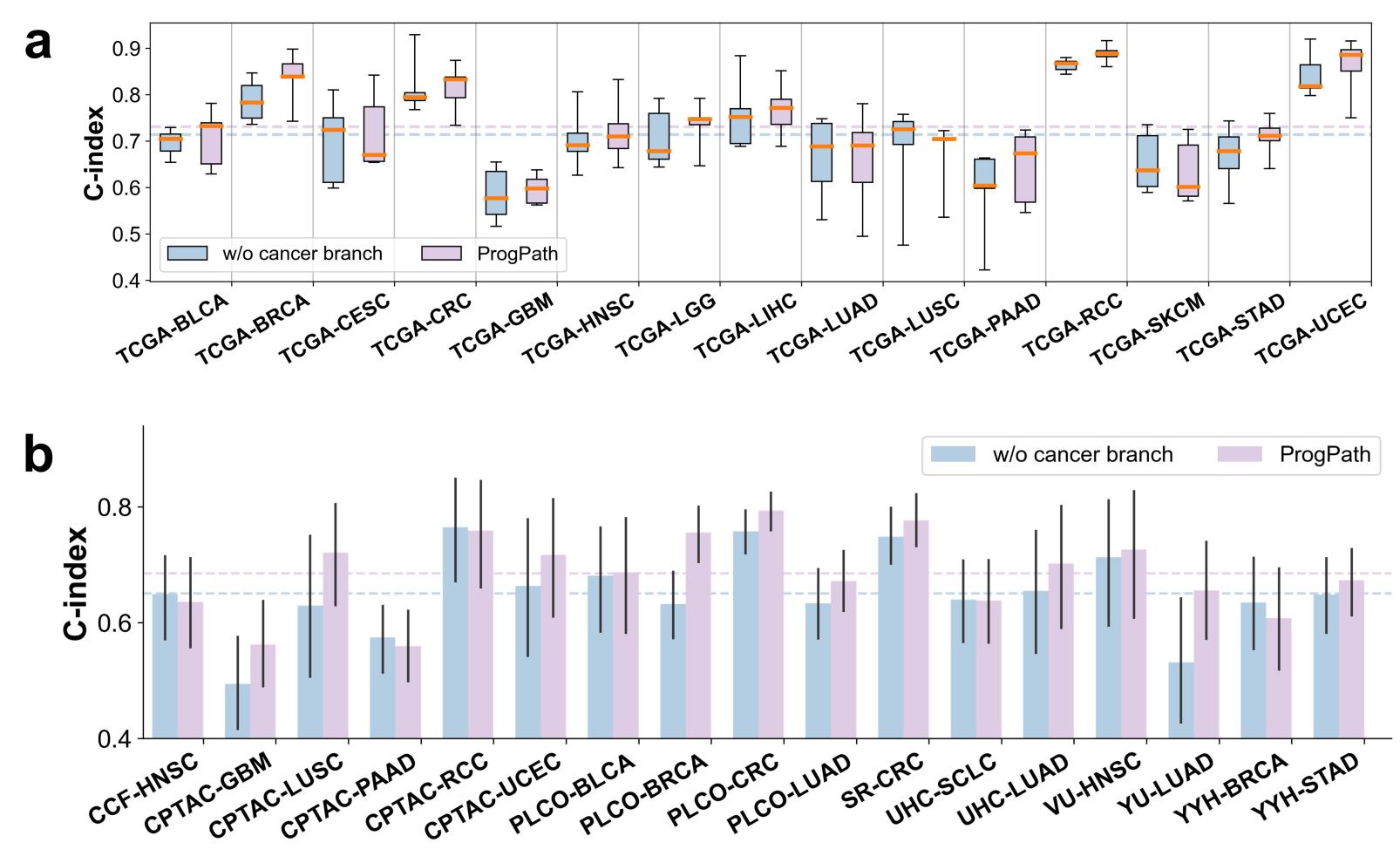


**Supplementary Figure 8: Ablation study of the effectiveness of cancer branch.** We ablate the effectiveness of newly presented cancer branch by comparing PROGPATH with a variant without cancer branch (including cancer encoder, cancer tokens and dynamic router). **a** Box plots of comparison between PROGPATH and PROGPATH without cancer branch on TCGA held-out sets. Overall, PROGPATH has a C-index performance gain of +1.7% compared to PROGPATH variant without cancer branch. **b** Bar plots of comparison between PROGPATH and PROGPATH without cancer branch on external datasets (PLCO, CPTAC and independent clinical cohorts). Overall, PROGPATH has a C-index performance gain of +4.2% compared to PROGPATH variant without cancer branch. Detailed numeric results are shown in Supplementary Table 6.

**Figure. S9.**


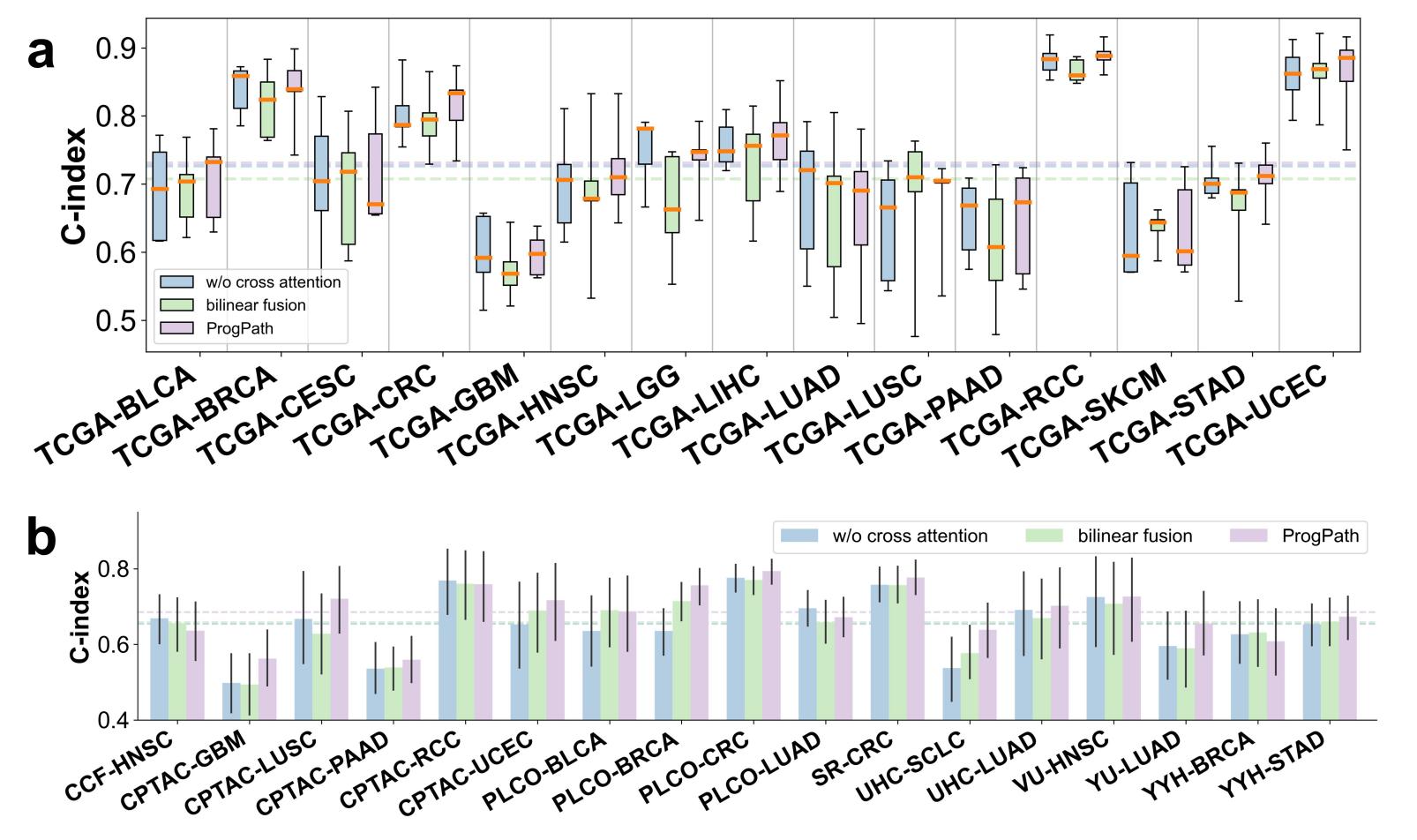


**Supplementary Figure 9: Ablation study of fusion method.** We evaluate the effectiveness of the fusion method by training PROGPATH models without Transformer-based cross attention and with bilinear fusion (instead of concatenation) separately. The results are presented in: **a** Box plots of comparison among different fusion methods on TCGA held-out sets. Overall, PROGPATH has a C-index performance gain of +0.1% and +2.2% compared to PROGPATH variant without cross attention and PROGPATH variant with bilinear fusion. **b** Bar plots of comparison among different fusion methods on external datasets (PLCO, CPTAC and independent clinical cohorts). Overall, PROGPATH has a C-index performance gain of +3.7% and +2.2% compared to PROGPATH variant without cross attention and PROGPATH variant with bilinear fusion. Detailed numeric results are shown in Supplementary Table 7.

**Figure. S10.**


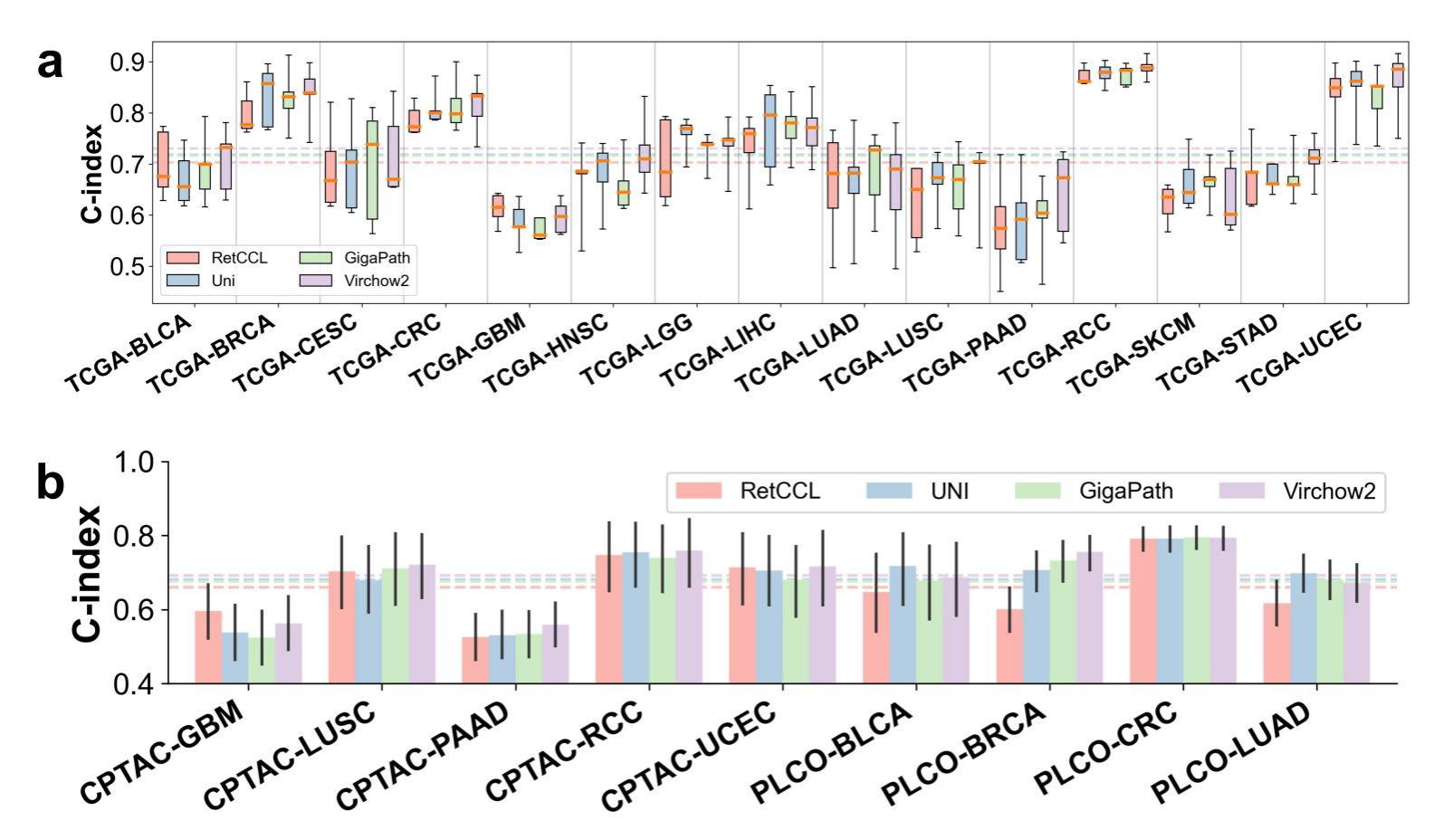


**Supplementary Figure 10: Ablation study of impact of different feature extractors.** We conducted experiments on four different widely used feature extractors: RetCCL3, UNI4, GigaPath5, and Virchow26. **a** Box plots of comparison among different feature extractors on TCGA held-out sets. Overall, Virchow2 has a C-index performance gain of +3.1%, +1.4% and +1.5% compared to RetCCL, UNI and GigaPath. **b** Bar plots of comparison among different feature extractors on external datasets (PLCO and CPTAC cohorts). Overall, Virchow2 has a C-index performance gain of +4.8%, +1.7% and +2.4% compared to RetCCL, UNI and GigaPath. Detailed numeric results are shown in Supplementary Table 8.

**Figure. S11.**


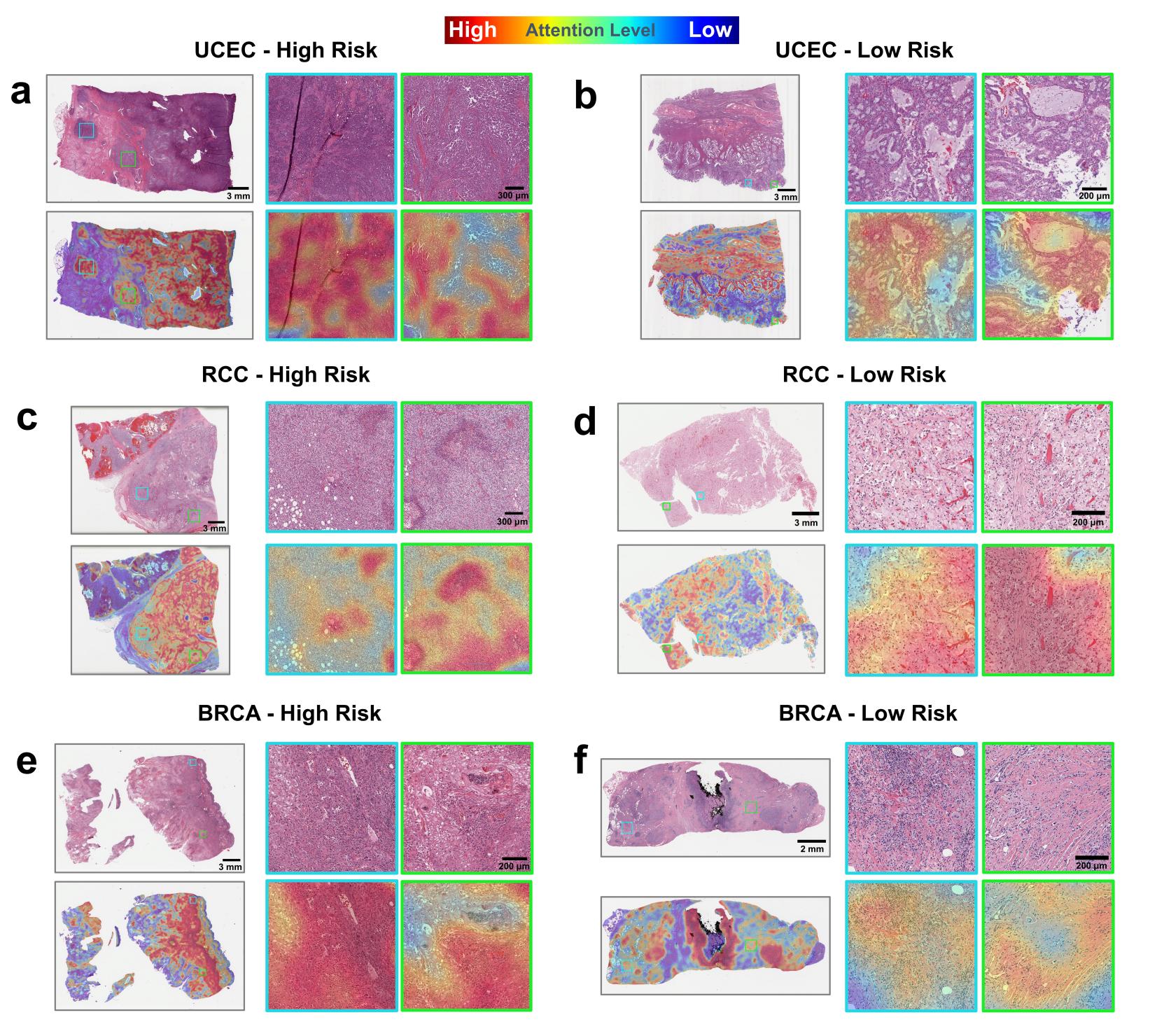


**Supplementary Figure 11: Representative heatmap visualization of UCEC, RCC, and BRCA.** Original whole slide images and information-rich patches, along with their corresponding attention heatmaps, are shown in each subfigure. These examples include a high-risk patient with UCEC (**a**) and a low-risk patient with UCEC (**b**), a high-risk patient with RCC (**c**) and a low-risk patient with RCC (**d**), and a high-risk patient with BRCA (**e**) and a low-risk patient with BRCA (**f**). Patches with low attention are outlined in blue, while patches with high attention are outlined in red.

**Table S1.**

**Supplementary Table 1:** **Patient characteristics of our study cohorts.**

| Dataset | Num.Slide  (Patient) | Age | Sex | | White | Asian | Black | Race  Indian | Islander | Other | NA | Disease Stage(Slide/Patient) | | | |
| --- | --- | --- | --- | --- | --- | --- | --- | --- | --- | --- | --- | --- | --- | --- | --- |
|  |  |  | Male | Female |  |  |  |  |  |  |  |  |  |  |  |
|  |  |  |  |  |  |  |  |  |  |  |  | Stage 0/1 | Stage 2 | Stage 3 | Stage 4 |
| TCGA-BLCA | 440(368) | 67.9±10.7 | 273 | 95 | 289 | 42 | 21 | / | / | / | 16 | 3/2 | 156/120 | 139/124 | 142/122 |
| TCGA-BRCA | 1124(1007) | 58.4±13.2 | 12 | 995 | 701 | 60 | 161 | 1 | / | / | 84 | 184/168 | 660/592 | 261/229 | 19/18 |
| TCGA-CESC | 275(258) | 48.2±13.4 | / | 258 | 177 | 19 | 25 | 4 | / | / | 33 | / | / | / | / |
| TCGA-CRC | 551(526) | 65.8±12.9 | 268 | 258 | 244 | 12 | 63 | 1 | / | / | 206 | 103/98 | 207/196 | 162/155 | 79/77 |
| TCGA-GBM | 789(349) | 56.7±14.4 | 214 | 135 | 134 | 1 | 16 | / | / | / | 198 | / | / | / | / |
| TCGA-HNSC | 397(378) | 61.0±11.7 | 274 | 104 | 318 | 9 | 37 | 1 | / | / | 13 | 25/22 | 68/63 | 68/67 | 236/226 |
| TCGA-RCC | 808(781) | 60.0±12.6 | 521 | 260 | 636 | 16 | 108 | 2 | / | / | 19 | 430/409 | 100/98 | 179/177 | 99/97 |
| TCGA-LGG | 833(480) | 43.1±13.4 | 266 | 214 | 444 | 6 | 20 | 1 | / | / | 9 | / | / | / | / |
| TCGA-LIHC | 340(332) | 58.8±13.3 | 226 | 106 | 157 | 154 | 11 | 2 | / | / | 8 | 166/162 | 85/83 | 84/82 | 5/5 |
| TCGA-LUAD | 470(419) | 64.8±10.2 | 193 | 226 | 328 | 8 | 49 | 1 | / | / | 33 | 248/239 | 119/105 | 81/53 | 22/22 |
| TCGA-LUSC | 430(405) | 67.1±8.6 | 303 | 102 | 287 | 8 | 25 | / | / | / | 85 | 221/203 | 133/131 | 70/65 | 6/6 |
| TCGA-PAAD | 193(173) | 64.7±11.1 | 96 | 77 | 152 | 11 | 6 | / | / | / | 4 | 20/20 | 160/144 | 4/4 | 9/5 |
| TCGA-SKCM | 393(350) | 57.3±15.8 | 218 | 132 | 334 | 10 | / | / | / | / | 6 | 68/67 | 107/102 | 195/161 | 23/20 |
| TCGA-STAD | 379(354) | 64.8±10.5 | 230 | 124 | 228 | 78 | 13 | / | / | / | 35 | 49/45 | 119/113 | 179/164 | 32/32 |
| TCGA-UCEC | 577(490) | 63.7±10.9 | / | 490 | 329 | 19 | 100 | 4 | 8 | / | 30 | 362/307 | 60/47 | 123/109 | 32/27 |
| CPTAC-GBM | 244(99) | 58.5±12.5 | 55 | 44 | 47 | 29 | / | / | / | 21 | 2 | / | / | / | / |
| CPTAC-LUSC | 292(103) | 66.5±8.4 | 84 | 19 | 68 | 23 | / | / | / | 11 | 1 | 97/37 | 127/44 | 65/21 | 3/1 |
| CPTAC-PAAD | 330(146) | 64.6±10.8 | 76 | 70 | 85 | 22 | 2 | / | / | 37 | / | 46/19 | 179/72 | 86/44 | 19/11 |
| CPTAC-RCC | 459(203) | 61.0±12.0 | 143 | 60 | 148 | 20 | 3 | / | / | 29 | 3 | 203/102 | 55/22 | 134/58 | 67/21 |
| CPTAC-UCEC | 546(227) | 64.9±9.8 | / | 227 | 174 | 2 | 6 | / | / | 42 | 3 | 388/163 | 47/20 | 92/34 | 19/10 |
| PLCO-BLCA | 466(276) | 63.8±4.8 | 216 | 60 | 262 | 8 | 3 | / | 2 | 1 | / | 366/230 | 70/34 | 12/5 | 18/7 |
| PLCO-BRCA | 1893(890) | 62.4±5.2 | / | 890 | 798 | 20 | 56 | / | 6 | 3 | 7 | 1124/577 | 652/271 | 95/35 | 22/7 |
| PLCO-CRC | 1269(656) | 64.7±5.3 | 380 | 276 | 584 | 20 | 33 | 2 | 10 | 2 | 5 | 397/211 | 348/181 | 393/200 | 131/64 |
| PLCO-LUAD | 176(176) | 76.4±6.8 | 85 | 91 | 158 | 3 | 8 | / | 0 | / | 7 | 121/121 | 18/18 | 27/27 | 10/10 |
| YYH-STAD | 265(265) | 64.3±9.0 | 201 | 64 | / | 265 | / | / | / | / | / | 75/75 | 81/81 | 106/106 | 3/3 |
| YYH-BRCA | 299(299) | 51.3±9.5 | / | 299 | / | 299 | / | / | / | / | / | 22/22 | 182/182 | 89/89 | 6/6 |
| CCF-HNSC | 322(322) | 58.7±9.1 | 285 | 37 | 299 | / | 19 | / | / | 4 | / | 155/155 | 86/86 | 81/81 | / |
| VU-HNSC | 137(137) | 55.8±8.6 | 126 | 11 | 133 | / | 4 | / | / | / | / | 99/99 | 29/29 | 9/9 | / |
| SR-CRC | 423(423) | 68.0±12.0 | 228 | 195 | / | / | / | / | / | / | 423 | 59/59 | 151/151 | 193/193 | 20/20 |
| YU-LUAD | 95(67) | 69.4±10.5 | 35 | 32 | / | / | / | / | / | / | 67 | / | / | 1/1 | 94/66 |
| UHC-LUAD | 62(56) | 62.4±11.1 | 23 | 33 | / | / | / | / | / | / | 56 | 10/8 | / | 10/9 | 42/39 |
| UHC-SCLC | 96(96) | 66.9±10.3 | 47 | 49 | / | / | / | / | / | / | 96 | / | 1/1 | 21/21 | 74/74 |

**Table S2.**

**Supplementary Table 2: Comparison of PROGPATH-H, PROGPATH-C and PROGPATH models.** The metrics evaluated include C-index, survival-AUC, and log-rank test’s significance for patient stratification (P value). The **bold** values denote the best performance in each row.

| Study | ProgPath-H | | | ProgPath-C | | |  | ProgPath | |
| --- | --- | --- | --- | --- | --- | --- | --- | --- | --- |
| TCGA-BLCA | C-index | AUC | P value | C-index | AUC | P value | C-index | AUC | P value |
|  | 0.657 | 0.668 | 6.28E-06 | 0.681 | 0.677 | 6.87E-07 | **0.707** | **0.717** | 3.40E-10 |
| TCGA-BRCA | 0.763 | 0.769 | 5.11E-10 | 0.775 | 0.781 | 6.99E-07 | **0.836** | **0.875** | 2.27E-09 |
| TCGA-CESC | 0.687 | 0.678 | 0.001 | 0.537 | 0.506 | 0.499 | **0.719** | **0.737** | 3.93E-06 |
| TCGA-CRC | 0.749 | 0.737 | 6.35E-07 | 0.765 | 0.761 | 6.00E-13 | **0.814** | **0.810** | 2.29E-12 |
| TCGS-GBM | 0.557 | 0.572 | 0.031 | **0.623** | 0.601 | 1.37E-04 | 0.596 | **0.610** | 1.07E-04 |
| TCGA-HNSC | 0.672 | 0.683 | 1.79E-05 | 0.595 | 0.618 | 0.005 | **0.721** | **0.712** | 9.48E-11 |
| TCGA-LGG | 0.696 | 0.683 | 1.10E-05 | **0.744** | 0.689 | 9.40E-11 | 0.734 | **0.702** | 1.37E-06 |
| TCGA-LIHC | 0.745 | 0.794 | 5.62E-07 | 0.698 | 0.698 | 8.69E-05 | **0.768** | **0.794** | 4.73E-06 |
| TCGA-LUAD | **0.678** | **0.681** | 3.83E-06 | 0.643 | 0.656 | 5.52E-06 | 0.659 | 0.658 | 1.66E-05 |
| TCGA-LUSC | 0.624 | 0.656 | 0.099 | 0.673 | 0.680 | 4.04E-04 | **0.674** | **0.704** | 5.11E-04 |
| TCGA-PAAD | **0.655** | **0.693** | 3.41E-04 | 0.557 | 0.549 | 0.092 | 0.644 | 0.671 | 7.70E-04 |
| TCGA-RCC | 0.828 | 0.836 | 2.03E-21 | 0.860 | 0.864 | 2.12E-26 | **0.888** | **0.898** | 2.42E-30 |
| TCGA-SKCM | 0.616 | 0.616 | 1.33E-04 | 0.613 | 0.612 | 0.043 | **0.634** | **0.631** | 2.97E-06 |
| TCGA-STAD | 0.688 | **0.701** | 2.05E-06 | 0.653 | 0.614 | 7.32E-04 | **0.708** | 0.663 | 1.53E-06 |
| TCGA-UCEC | 0.796 | 0.826 | 2.10E-09 | 0.822 | 0.806 | 3.35E-07 | **0.860** | **0.868** | 2.63E-11 |
| TCGA-avg | 0.694 | 0.706 | / | 0.683 | 0.674 | / | **0.731** | **0.737** | / |
| PLCO-BLCA | 0.637 | 0.640 | 0.040 | 0.650 | 0.646 | 0.113 | **0.687** | **0.692** | 0.032 |
| PLCO-BRCA | 0.661 | 0.666 | 3.16E-04 | 0.627 | 0.611 | 2.78E-05 | **0.756** | **0.751** | 1.85E-07 |
| PLCO-CRC | 0.718 | 0.735 | 2.47E-12 | 0.787 | 0.810 | 2.24E-20 | **0.794** | **0.815** | 6.29E-23 |
| PLCO-LUAD | 0.611 | 0.645 | 0.011 | 0.597 | 0.622 | 0.098 | **0.672** | **0.721** | 7.27E-04 |
| PLCO-avg | 0.657 | 0.671 | / | 0.665 | 0.672 | / | **0.727** | **0.745** | / |
| CPTAC-GBM | 0.444 | 0.419 | 0.063 | **0.596** | **0.624** | 0.051 | 0.563 | 0.592 | 0.149 |
| CPTAC-LUSC | 0.665 | 0.712 | 0.002 | 0.645 | 0.668 | 0.034 | **0.721** | **0.765** | 0.017 |
| CPTAC-PAAD | 0.537 | 0.545 | 0.151 | 0.506 | 0.516 | 0.469 | **0.560** | **0.576** | 0.102 |
| CPTAC-RCC | 0.670 | 0.686 | 0.037 | **0.776** | **0.792** | 2.46E-05 | 0.759 | 0.779 | 2.46E-05 |
| CPTAC-UCEC | 0.580 | 0.609 | 0.194 | **0.738** | 0.745 | 1.06E-04 | 0.717 | **0.745** | 0.009 |
| CPTAC-avg | 0.579 | 0.594 | / | 0.652 | 0.669 | / | **0.664** | **0.691** | / |
| CCF-HNSC | 0.624 | 0.628 | 0.018 | 0.635 | **0.636** | 0.320 | **0.636** | 0.628 | 0.089 |
| VU-HNSC | 0.705 | 0.719 | 0.215 | 0.544 | 0.557 | 0.689 | **0.727** | **0.752** | 0.019 |
| YYH-STAD | 0.644 | 0.670 | 3.62E-4 | **0.697** | **0.740** | 3.73E-07 | 0.673 | 0.712 | 5.11E-06 |
| YYH-BRCA | 0.588 | 0.575 | 0.045 | 0.598 | 0.569 | 0.157 | **0.608** | **0.590** | 0.005 |
| SR-CRC | 0.756 | 0.782 | 1.29E-12 | 0.702 | 0.687 | 5.67E-09 | **0.777** | **0.798** | 2.50E-10 |
| YU-LUAD | 0.575 | 0.612 | 0.071 | 0.579 | 0.608 | 0.055 | **0.656** | **0.716** | 0.039 |
| UHC-LUAD | 0.585 | 0.617 | 0.084 | 0.680 | 0.746 | 0.102 | **0.702** | **0.749** | 0.005 |
| UHC-SCLC | 0.514 | 0.519 | 0.373 | 0.626 | 0.664 | 0.004 | **0.638** | **0.670** | 0.058 |
| IS-avg | 0.624 | 0.640 | / | 0.633 | 0.651 | / | **0.677** | **0.702** | / |

**Table S3**

**Supplementary Table 3:** **Comparison results between the standard Cox model with age, sex and tumor stage as input and PROGPATH.** The metrics evaluated include C-index, survival-AUC, and log-rank test’s significance for patient stratification (P value). The **bold** values denote the best performance in each row.

| Study | Cox model | | |  | ProgPath | |
| --- | --- | --- | --- | --- | --- | --- |
| TCGA-BLCA | C-index | AUC | P value | C-index | AUC | P value |
|  | 0.639 | 0.632 | 1.08E-04 | **0.707** | **0.717** | 3.40E-10 |
| TCGA-BRCA | 0.778 | 0.828 | 3.19E-08 | **0.836** | **0.875** | 2.27E-09 |
| TCGA-CESC | 0.524 | 0.480 | 0.813 | **0.719** | **0.737** | 3.93E-06 |
| TCGA-CRC | 0.766 | 0.788 | 9.43E-12 | **0.814** | **0.810** | 2.29E-12 |
| TCGS-GBM | **0.615** | **0.653** | 1.69E-08 | 0.596 | 0.610 | 1.07E-04 |
| TCGA-HNSC | 0.617 | 0.599 | 3.26E-04 | **0.721** | **0.712** | 9.48E-11 |
| TCGA-LGG | **0.782** | **0.831** | 3.92E-14 | 0.734 | 0.702 | 1.37E-06 |
| TCGA-LIHC | 0.677 | 0.700 | 0.001 | **0.768** | **0.794** | 4.73E-06 |
| TCGA-LUAD | 0.663 | 0.671 | 6.16E-11 | **0.659** | **0.658** | 1.66E-05 |
| TCGA-LUSC | 0.673 | 0.672 | 7.16E-06 | **0.674** | **0.704** | 0.001 |
| TCGA-PAAD | 0.477 | 0.445 | 0.503 | **0.644** | **0.671** | 0.001 |
| TCGA-RCC | 0.863 | 0.872 | 1.96E-26 | **0.888** | **0.898** | 2.42E-30 |
| TCGA-SKCM | 0.547 | 0.579 | 0.989 | **0.634** | **0.631** | 2.97E-06 |
| TCGA-STAD | 0.640 | 0.644 | 4.98E-04 | **0.708** | **0.663** | 1.53E-06 |
| TCGA-UCEC | 0.776 | 0.790 | 1.42E-06 | **0.860** | **0.868** | 2.63E-11 |
| TCGA-avg | 0.669 | 0.679 | / | **0.731** | **0.737** | / |
| PLCO-BLCA | 0.230 | 0.194 | 2.21E-10 | **0.687** | **0.692** | 0.032 |
| PLCO-BRCA | 0.183 | 0.140 | 1.06E-29 | **0.756** | **0.751** | 1.85E-07 |
| PLCO-CRC | 0.589 | 0.629 | 0.007 | **0.794** | **0.815** | 6.29E-23 |
| PLCO-LUAD | **0.755** | **0.843** | 2.89E-47 | 0.672 | 0.721 | 0.001 |
| PLCO-avg | 0.439 | 0.452 | / | **0.727** | **0.745** | / |
| CPTAC-GBM | **0.577** | 0.588 | 0.002 | 0.563 | **0.592** | 0.149 |
| CPTAC-LUSC | 0.369 | 0.397 | 2.50E-04 | **0.721** | **0.765** | 0.017 |
| CPTAC-PAAD | 0.510 | 0.515 | 0.983 | **0.560** | **0.576** | 0.102 |
| CPTAC-RCC | 0.634 | 0.616 | 0.010 | **0.759** | **0.779** | 2.46E-05 |
| CPTAC-UCEC | 0.394 | 0.394 | 0.195 | **0.717** | **0.745** | 0.009 |
| CPTAC-avg | 0.497 | 0.502 | / | **0.664** | **0.691** | / |
| CCF-HNSC | 0.571 | 0.595 | 0.209 | **0.636** | **0.628** | 0.089 |
| VU-HNSC | 0.522 | 0.536 | 0.220 | **0.727** | **0.752** | 0.019 |
| YYH-STAD | **0.687** | 0.699 | 2.79E-06 | 0.673 | **0.712** | 5.11E-06 |
| YYH-BRCA | 0.503 | 0.486 | 0.741 | **0.608** | **0.590** | 0.005 |
| SR-CRC | 0.570 | 0.423 | 0.028 | **0.777** | **0.798** | 2.50E-10 |
| YU-LUAD | 0.495 | 0.510 | 0.469 | **0.656** | **0.716** | 0.039 |
| UHC-LUAD | 0.402 | 0.372 | 0.492 | **0.702** | **0.749** | 0.005 |
| UHC-SCLC | 0.439 | 0.418 | 0.245 | **0.638** | **0.670** | 0.058 |
| IS-avg | 0.524 | 0.505 | / | **0.677** | **0.702** | / |

**Table S4.**

**Supplementary Table 4:** **Comparison of MCAT, MOTCAT, SurvPath and PROGPATH models.** The metrics evaluated include C-index, survival-AUC, and log-rank test’s significance for patient stratification (P value). The **bold** values denote the best performance in each row.

| Study |  | MCAT |  |  | MOTCAT | |  | SurvPath | |  | ProgPath | |
| --- | --- | --- | --- | --- | --- | --- | --- | --- | --- | --- | --- | --- |
|  | C-index | AUC | P value | C-index | AUC | P value | C-index | AUC | P value | C-index | AUC | P value |
| TCGA-BLCA | 0.642 | 0.651 | 6.13E-05 | 0.673 | 0.674 | 6.02E-06 | 0.641 | 0.649 | 2.96E-06 | **0.707** | **0.717** | 3.40E-10 |
| TCGA-BRCA | 0.758 | 0.717 | 5.21E-07 | 0.763 | 0.790 | 2.11E-07 | 0.737 | 0.729 | 1.13E-05 | **0.836** | **0.875** | 2.27E-09 |
| TCGA-CESC | 0.706 | 0.698 | 1.33E-03 | 0.706 | 0.696 | 0.007 | 0.712 | 0.728 | 0.018 | **0.719** | **0.737** | 3.93E-06 |
| TCGA-CRC | 0.761 | 0.755 | 5.73E-09 | 0.772 | 0.735 | 4.79E-10 | 0.715 | 0.706 | 1.29E-04 | **0.814** | **0.810** | 2.29E-12 |
| TCGS-GBM | 0.557 | 0.537 | 0.045 | 0.547 | 0.553 | 0.118 | 0.570 | 0.574 | 0.004 | **0.596** | **0.610** | 1.07E-04 |
| TCGA-HNSC | 0.669 | 0.664 | 2.74E-05 | 0.680 | 0.688 | 0.001 | 0.699 | 0.709 | 2.64E-06 | **0.721** | **0.712** | 9.48E-11 |
| TCGA-LGG | 0.695 | 0.703 | 1.46E-05 | 0.706 | **0.719** | 3.87E-06 | 0.680 | 0.690 | 5.67E-04 | **0.734** | 0.702 | 1.37E-06 |
| TCGA-LIHC | 0.740 | 0.762 | 1.04E-04 | 0.761 | 0.738 | 1.01E-05 | 0.722 | 0.744 | 7.54E-04 | **0.768** | **0.794** | 4.73E-06 |
| TCGA-LUAD | 0.614 | 0.593 | 3.75E-04 | 0.634 | 0.640 | 3.85E-05 | 0.625 | 0.648 | 0.002 | **0.659** | **0.658** | 1.66E-05 |
| TCGA-LUSC | 0.625 | 0.617 | 0.022 | 0.618 | 0.603 | 0.024 | 0.630 | 0.628 | 0.022 | **0.674** | **0.704** | 5.11E-04 |
| TCGA-PAAD | 0.577 | 0.594 | 0.072 | 0.613 | 0.634 | 0.051 | 0.609 | 0.631 | 0.036 | **0.644** | **0.671** | 7.7E-04 |
| TCGA-RCC | 0.822 | 0.810 | 1.51E-20 | 0.821 | 0.815 | 3.23E-23 | 0.794 | 0.813 | 1.37E-20 | **0.888** | **0.898** | 2.42E-30 |
| TCGA-SKCM | 0.621 | 0.620 | 2.48E-04 | 0.595 | 0.592 | 0.007 | **0.634** | **0.663** | 5.19E-05 | 0.634 | 0.631 | 2.97E-06 |
| TCGA-STAD | 0.652 | 0.655 | 1.40E-05 | 0.645 | 0.662 | 5.80E-05 | 0.618 | 0.613 | 4.44E-04 | **0.708** | **0.663** | 1.53E-06 |
| TCGA-UCEC | 0.811 | 0.790 | 8.28E-11 | 0.806 | 0.783 | 3.10E-11 | 0.789 | 0.814 | 2.41E-08 | **0.860** | **0.868** | 2.63E-11 |
| TCGA-avg | 0.683 | 0.678 | / | 0.689 | 0.688 | / | 0.678 | 0.689 | / | **0.731** | **0.737** | / |
| PLCO-BLCA | 0.631 | 0.631 | 0.066 | 0.622 | 0.634 | 0.348 | 0.648 | 0.644 | 0.029 | **0.687** | **0.692** | 0.032 |
| PLCO-BRCA | 0.646 | 0.648 | 0.001 | 0.610 | 0.610 | 5.85E-04 | 0.636 | 0.638 | 0.007 | **0.756** | **0.751** | 1.85E-07 |
| PLCO-CRC | 0.727 | 0.735 | 3.99E-12 | 0.721 | 0.730 | 1.68E-16 | 0.683 | 0.687 | 5.36E-09 | **0.794** | **0.815** | 6.29E-23 |
| PLCO-LUAD | 0.622 | 0.669 | 3.27E-04 | 0.609 | 0.652 | 1.66E-04 | 0.649 | 0.695 | 1.39E-05 | **0.672** | **0.721** | 7.27E-04 |
| PLCO-avg | 0.656 | 0.671 | / | 0.640 | 0.657 | / | 0.654 | 0.666 | / | **0.727** | **0.745** | / |
| CPTAC-GBM | 0.520 | 0.535 | 0.833 | 0.505 | 0.499 | 0.802 | 0.421 | 0.383 | 0.096 | **0.563** | **0.592** | 0.149 |
| CPTAC-LUSC | 0.610 | 0.621 | 0.041 | 0.615 | 0.630 | 0.280 | 0.625 | 0.658 | 0.057 | **0.721** | **0.765** | 0.017 |
| CPTAC-PAAD | 0.517 | 0.514 | 0.723 | **0.576** | **0.597** | 0.040 | 0.568 | 0.586 | 0.034 | 0.560 | 0.576 | 0.102 |
| CPTAC-RCC | 0.653 | 0.660 | 4.81E-03 | 0.626 | 0.638 | 0.126 | 0.666 | 0.685 | 0.048 | **0.759** | **0.779** | 2.46E-05 |
| CPTAC-UCEC | 0.623 | 0.669 | 0.022 | 0.658 | 0.694 | 0.067 | 0.584 | 0.630 | 0.103 | **0.717** | **0.745** | 0.009 |
| CPTAC-avg | 0.585 | 0.600 | / | 0.596 | 0.612 | / | 0.573 | 0.588 | / | **0.664** | **0.691** | / |
| CCF-HNSC | 0.610 | 0.596 | 0.096 | 0.627 | 0.610 | 0.048 | **0.645** | 0.621 | 0.038 | 0.636 | **0.628** | 0.089 |
| VU-HNSC | 0.723 | 0.749 | 0.005 | 0.720 | 0.744 | 0.028 | 0.714 | 0.732 | 0.020 | **0.727** | **0.752** | 0.019 |
| YYH-STAD | 0.644 | 0.653 | 2.08E-04 | 0.653 | 0.663 | 3.40E-04 | 0.628 | 0.637 | 0.002 | **0.673** | **0.712** | 5.11E-06 |
| YYH-BRCA | 0.536 | 0.513 | 0.402 | 0.592 | 0.581 | 0.002 | 0.602 | 0.580 | 0.002 | **0.608** | **0.590** | 0.005 |
| SR-CRC | 0.692 | 0.686 | 7.11E-09 | 0.667 | 0.686 | 8.91E-06 | 0.694 | 0.721 | 3.25E-07 | **0.777** | **0.798** | 2.50E-10 |
| YU-LUAD | 0.533 | 0.565 | 0.598 | 0.551 | 0.593 | 0.268 | 0.580 | 0.620 | 0.515 | **0.656** | **0.716** | 0.039 |
| UHC-LUAD | 0.544 | 0.552 | 0.970 | 0.556 | 0.562 | 0.927 | 0.595 | 0.612 | 0.183 | **0.702** | **0.749** | 0.005 |
| UHC-SCLC | 0.584 | 0.603 | 0.094 | 0.599 | 0.631 | 0.050 | 0.571 | 0.583 | 0.228 | **0.638** | **0.670** | 0.058 |
| IS-avg | 0.608 | 0.615 | / | 0.621 | 0.634 | / | 0.629 | 0.638 | / | **0.677** | **0.702** | / |

**Table S5.**

**Supplementary Table 5: Comparison of PROGPATH and the results reported in the literature in the TCGA database.** The evaluation metric is C-index.

| Dataset | Survpath | MCAT | MOTCAT | PORPOISE | ProgPath |
| --- | --- | --- | --- | --- | --- |
| BLCA | 0.625 | 0.624 | 0.683 | 0.631 | **0.707** |
| BRCA | 0.655 | 0.580 | 0.673 | 0.628 | **0.836** |
| CESC | / | / | / | / | **0.719** |
| CRC | 0.673 | / | / | 0.640 | **0.814** |
| HNSC | 0.600 | / | / | 0.573 | **0.721** |
| LGG | / | / | / | **0.808** | 0.734 |
| LIHC | / | / | / | 0.622 | **0.768** |
| LUAD | / | 0.620 | **0.670** | 0.600 | 0.659 |
| LUSC | / | / | / | 0.538 | **0.674** |
| PAAD | / | / | / | / | **0.644** |
| RCC | / | / | / | / | **0.888** |
| SKCM | / | / | / | **0.651** | 0.634 |
| STAD | 0.592 | / | / | 0.563 | **0.708** |
| UCEC | / | 0.622 | 0.675 | 0.634 | **0.860** |

**Table S6.**

**Supplementary Table 6: Ablation study of effectiveness of cancer branch.** The metrics evaluated include C-index, survival-AUC, and log-rank test’s significance for patient stratification (P value). The **bold** values denote the best performance in each row.

| Study | w/o cancer branch | | |  | ProgPath | |
| --- | --- | --- | --- | --- | --- | --- |
| TCGA-BLCA | C-index | AUC | P value | C-index | AUC | P value |
|  | 0.696 | 0.712 | 2.74E-06 | **0.707** | **0.717** | 3.40E-10 |
| TCGA-BRCA | 0.787 | 0.782 | 1.01E-06 | **0.836** | **0.875** | 2.27E-09 |
| TCGA-CESC | 0.699 | 0.683 | 0.002 | **0.719** | **0.737** | 3.93E-06 |
| TCGA-CRC | **0.817** | 0.804 | 4.47E-09 | 0.814 | **0.810** | 2.29E-12 |
| TCGS-GBM | 0.585 | 0.567 | 0.019 | **0.596** | **0.610** | 1.07E-04 |
| TCGA-HNSC | 0.704 | 0.686 | 2.57E-09 | **0.721** | **0.712** | 9.48E-11 |
| TCGA-LGG | 0.707 | 0.676 | 1.04E-04 | **0.734** | **0.702** | 1.37E-06 |
| TCGA-LIHC | 0.758 | 0.736 | 2.37E-05 | **0.768** | **0.794** | 4.73E-06 |
| TCGA-LUAD | **0.664** | **0.665** | 7.39E-08 | 0.659 | 0.658 | 1.66E-05 |
| TCGA-LUSC | **0.679** | 0.688 | 4.08E-04 | 0.674 | **0.704** | 5.11E-04 |
| TCGA-PAAD | 0.590 | 0.599 | 0.016 | **0.644** | **0.671** | 7.70E-04 |
| TCGA-RCC | 0.864 | 0.862 | 8.78E-28 | **0.888** | **0.898** | 2.42E-30 |
| TCGA-SKCM | **0.655** | **0.679** | 1.71E-05 | 0.634 | 0.631 | 2.97E-06 |
| TCGA-STAD | 0.667 | **0.697** | 5.21E-05 | **0.708** | 0.663 | 1.53E-06 |
| TCGA-UCEC | 0.843 | 0.834 | 3.93E-10 | **0.860** | **0.868** | 2.63E-11 |
| TCGA-avg | 0.714 | 0.711 | / | **0.731** | **0.737** | / |
| PLCO-BLCA | 0.682 | 0.690 | 0.319 | **0.687** | **0.692** | 0.032 |
| PLCO-BRCA | 0.632 | 0.644 | 0.004 | **0.756** | **0.751** | 1.85E-07 |
| PLCO-CRC | 0.758 | 0.773 | 5.82E-16 | **0.794** | **0.815** | 6.29E-23 |
| PLCO-LUAD | 0.634 | 0.672 | 0.005 | **0.672** | **0.721** | 7.27E-04 |
| PLCO-avg | 0.676 | 0.695 | / | **0.727** | **0.745** | / |
| CPTAC-GBM | 0.495 | 0.507 | 0.908 | **0.563** | **0.592** | 0.149 |
| CPTAC-LUSC | 0.630 | 0.628 | 0.032 | **0.721** | **0.765** | 0.017 |
| CPTAC-PAAD | **0.575** | **0.586** | 0.007 | 0.560 | 0.576 | 0.102 |
| CPTAC-RCC | **0.765** | **0.788** | 1.77E-04 | 0.759 | 0.779 | 2.46E-05 |
| CPTAC-UCEC | 0.663 | 0.694 | 0.086 | **0.717** | **0.745** | 0.009 |
| CPTAC-avg | 0.626 | 0.641 | / | **0.664** | **0.691** | / |
| CCF-HNSC | **0.649** | **0.635** | 0.002 | 0.636 | 0.628 | 0.089 |
| VU-HNSC | 0.713 | 0.740 | 0.005 | **0.727** | **0.752** | 0.019 |
| YYH-STAD | 0.648 | 0.657 | 7.13E-04 | **0.673** | **0.712** | 5.11E-06 |
| YYH-BRCA | **0.635** | **0.623** | 4.42E-04 | 0.608 | 0.590 | 0.005 |
| SR-CRC | 0.749 | 0.769 | 1.01E-09 | **0.777** | **0.798** | 2.50E-10 |
| YU-LUAD | 0.531 | 0.554 | 0.516 | **0.656** | **0.716** | 0.039 |
| UHC-LUAD | 0.655 | 0.705 | 0.028 | **0.702** | **0.749** | 0.005 |
| UHC-SCLC | **0.640** | **0.682** | 9.78E-04 | 0.638 | 0.670 | 0.058 |
| IS-avg | 0.653 | 0.670 | / | **0.677** | **0.702** | / |

**Table S7.**

**Supplementary Table 7: Ablation study of effectiveness of fusion method.** The metrics evaluated include C-index, survival-AUC, and log-rank test’s significance for patient stratification (P value). The **bold** values denote the best performance in each row.

| Study | w/o cross attention | | | Bilinear fusion | | |  | ProgPath | |
| --- | --- | --- | --- | --- | --- | --- | --- | --- | --- |
| TCGA-BLCA | C-index | AUC | P value | C-index | AUC | P value | C-index | AUC | P value |
|  | 0.689 | 0.672 | 2.02E-08 | 0.692 | 0.690 | 3.32E-06 | **0.707** | **0.717** | 3.40E-10 |
| TCGA-BRCA | **0.839** | 0.863 | 2.12E-11 | 0.818 | 0.846 | 2.86E-10 | 0.836 | **0.875** | 2.27E-09 |
| TCGA-CESC | 0.704 | 0.698 | 9.99E-05 | 0.694 | 0.663 | 1.66E-05 | **0.719** | **0.737** | 3.93E-06 |
| TCGA-CRC | 0.805 | 0.805 | 5.11E-14 | 0.793 | 0.789 | 2.82E-09 | **0.814** | **0.810** | 2.29E-12 |
| TCGS-GBM | **0.597** | 0.600 | 3.01E-04 | 0.574 | 0.576 | 4.51E-05 | 0.596 | **0.610** | 1.07E-04 |
| TCGA-HNSC | 0.701 | 0.696 | 5.44E-09 | 0.684 | 0.679 | 1.09E-06 | **0.721** | **0.712** | 9.48E-11 |
| TCGA-LGG | **0.750** | **0.733** | 2.37E-07 | 0.666 | 0.658 | 3.77E-04 | 0.734 | 0.702 | 1.37E-06 |
| TCGA-LIHC | 0.759 | **0.804** | 6.19E-06 | 0.727 | 0.741 | 1.66E-04 | **0.768** | 0.794 | 4.73E-06 |
| TCGA-LUAD | **0.683** | **0.700** | 4.56E-05 | 0.660 | 0.651 | 1.27E-06 | 0.659 | 0.658 | 1.66E-05 |
| TCGA-LUSC | 0.641 | 0.663 | 3.28E-04 | **0.677** | 0.691 | 1.87E-04 | 0.674 | **0.704** | 5.11E-04 |
| TCGA-PAAD | **0.650** | **0.686** | 0.012 | 0.610 | 0.644 | 2.76E-04 | 0.644 | 0.671 | 7.70E-04 |
| TCGA-RCC | 0.883 | 0.888 | 1.74E-29 | 0.866 | 0.884 | 3.77E-28 | **0.888** | **0.898** | 2.42E-30 |
| TCGA-SKCM | 0.634 | **0.650** | 8.82E-05 | **0.634** | 0.637 | 0.001 | 0.634 | 0.631 | 2.97E-06 |
| TCGA-STAD | 0.706 | **0.736** | 1.66E-06 | 0.660 | 0.675 | 3.42E-05 | **0.708** | 0.663 | 1.53E-06 |
| TCGA-UCEC | 0.858 | **0.877** | 2.44E-10 | **0.862** | 0.866 | 6.93E-09 | 0.860 | 0.868 | 2.63E-11 |
| TCGA-avg | 0.727 | **0.738** | / | 0.708 | 0.713 | / | **0.731** | 0.737 | / |
| PLCO-BLCA | 0.636 | 0.641 | 0.069 | **0.690** | **0.695** | 0.025 | 0.687 | 0.692 | 0.032 |
| PLCO-BRCA | 0.635 | 0.621 | 0.006 | 0.715 | 0.716 | 1.14E-07 | **0.756** | **0.751** | 1.85E-07 |
| PLCO-CRC | 0.776 | 0.792 | 5.49E-17 | 0.770 | 0.789 | 5.37E-19 | **0.794** | **0.815** | 6.29E-23 |
| PLCO-LUAD | 0.695 | **0.757** | 5.11E-08 | 0.658 | 0.704 | 2.52E-05 | **0.672** | 0.721 | 7.27E-04 |
| PLCO-avg | 0.686 | 0.703 | / | 0.708 | 0.726 | / | **0.727** | **0.745** | / |
| CPTAC-GBM | 0.498 | 0.497 | 0.935 | 0.493 | 0.491 | 0.963 | **0.563** | **0.592** | 0.149 |
| CPTAC-LUSC | 0.668 | 0.708 | 0.001 | 0.628 | 0.636 | 0.104 | **0.721** | **0.765** | 0.017 |
| CPTAC-PAAD | 0.535 | 0.545 | 0.050 | 0.539 | 0.553 | 0.415 | **0.560** | **0.576** | 0.102 |
| CPTAC-RCC | **0.769** | **0.786** | 2.03E-05 | 0.761 | 0.782 | 1.05E-05 | 0.759 | 0.779 | 2.46E-05 |
| CPTAC-UCEC | 0.653 | 0.673 | 0.118 | 0.690 | 0.726 | 0.073 | **0.717** | **0.745** | 0.009 |
| CPTAC-avg | 0.625 | 0.642 | / | 0.622 | 0.637 | / | **0.664** | **0.691** | / |
| CCF-HNSC | **0.669** | **0.673** | 0.001 | 0.657 | 0.642 | 0.022 | 0.636 | 0.628 | 0.089 |
| VU-HNSC | 0.725 | 0.741 | 0.005 | 0.707 | 0.726 | 0.013 | **0.727** | **0.752** | 0.019 |
| YYH-STAD | 0.653 | 0.700 | 4.34E-05 | 0.660 | 0.677 | 8.08E-05 | **0.673** | **0.712** | 5.11E-06 |
| YYH-BRCA | 0.627 | 0.594 | 0.004 | **0.631** | **0.600** | 0.026 | 0.608 | 0.590 | 0.005 |
| SR-CRC | 0.758 | **0.812** | 2.32E-09 | 0.757 | 0.691 | 1.56E-11 | **0.777** | 0.798 | 2.50E-10 |
| YU-LUAD | 0.596 | 0.617 | 0.097 | 0.590 | 0.625 | 0.022 | **0.656** | **0.716** | 0.039 |
| UHC-LUAD | 0.691 | 0.745 | 0.059 | 0.669 | 0.737 | 0.071 | **0.702** | **0.749** | 0.005 |
| UHC-SCLC | 0.538 | 0.559 | 0.260 | 0.577 | 0.604 | 0.149 | **0.638** | **0.670** | 0.058 |
| IS-avg | 0.657 | 0.680 | / | 0.656 | 0.663 | / | **0.677** | **0.702** | / |

**Table S8.**

**Supplementary Table 8:** **Ablation study of impact of different feature extractors.** The metrics evaluated include C-index, survival-AUC, and log-rank test’s significance for patient stratification (P value). The **bold** values denote the best performance in each row.

| Study |  | Retccl |  |  | UNI |  |  | GigaPath | |  | ProgPath | |
| --- | --- | --- | --- | --- | --- | --- | --- | --- | --- | --- | --- | --- |
|  | C-index | AUC | P value | C-index | AUC | P value | C-index | AUC | P value | C-index | AUC | P value |
| TCGA-BLCA | 0.699 | 0.684 | 3.78E-07 | 0.671 | 0.667 | 6.88E-06 | 0.692 | 0.677 | 5.32E-07 | **0.707** | **0.717** | 3.40E-10 |
| TCGA-BRCA | 0.799 | 0.838 | 7.68E-06 | 0.834 | 0.834 | 4.03E-10 | 0.829 | 0.866 | 2.08E-10 | **0.836** | **0.875** | 2.27E-09 |
| TCGA-CESC | 0.691 | 0.691 | 5.66E-05 | 0.696 | 0.704 | 5.81E-04 | 0.698 | 0.674 | 6.72E-04 | **0.719** | **0.737** | 3.93E-06 |
| TCGA-CRC | 0.786 | 0.797 | 5.66E-10 | 0.810 | 0.807 | 1.74E-11 | **0.815** | **0.810** | 4.34E-12 | 0.814 | **0.810** | 2.29E-12 |
| TCGS-GBM | **0.612** | **0.622** | 8.23E-05 | 0.585 | 0.554 | 2.17E-04 | 0.572 | 0.567 | 5.84E-04 | 0.596 | 0.610 | 1.07E-04 |
| TCGA-HNSC | 0.665 | 0.657 | 7.64E-08 | 0.681 | 0.668 | 1.30E-08 | 0.658 | 0.672 | 3.86E-06 | **0.721** | **0.712** | 9.48E-11 |
| TCGA-LGG | 0.704 | 0.719 | 1.80E-06 | **0.757** | **0.763** | 1.84E-07 | 0.729 | 0.755 | 4.60E-06 | 0.734 | 0.702 | 1.37E-06 |
| TCGA-LIHC | 0.731 | 0.757 | 5.95E-06 | 0.768 | 0.775 | 4.06E-07 | **0.772** | 0.793 | 5.71E-06 | 0.768 | **0.794** | 4.73E-06 |
| TCGA-LUAD | 0.660 | 0.657 | 5.62E-07 | 0.662 | **0.667** | 5.08E-08 | **0.686** | 0.650 | 1.80E-06 | 0.659 | 0.658 | 1.66E-05 |
| TCGA-LUSC | 0.624 | 0.657 | 0.024 | 0.666 | 0.653 | 8.46E-05 | 0.657 | 0.665 | 0.001 | **0.674** | **0.704** | 5.11E-04 |
| TCGA-PAAD | 0.579 | 0.579 | 0.025 | 0.591 | 0.579 | 0.010 | 0.594 | 0.599 | 0.015 | **0.644** | **0.671** | 7.70E-04 |
| TCGA-RCC | 0.872 | 0.876 | 1.47E-29 | 0.877 | 0.887 | 1.30E-29 | 0.875 | 0.891 | 3.41E-28 | **0.888** | **0.898** | 2.42E-30 |
| TCGA-SKCM | 0.623 | 0.628 | 2.32E-05 | **0.664** | **0.716** | 1.36E-07 | 0.664 | 0.661 | 1.81E-07 | 0.634 | 0.631 | 2.97E-06 |
| TCGA-STAD | 0.675 | 0.654 | 6.99E-05 | 0.672 | 0.652 | 2.01E-04 | 0.675 | **0.669** | 2.06E-05 | **0.708** | 0.663 | 1.53E-06 |
| TCGA-UCEC | 0.830 | 0.842 | 1.34E-10 | 0.847 | 0.867 | 2.16E-09 | 0.829 | 0.844 | 2.58E-09 | **0.860** | **0.868** | 2.63E-11 |
| TCGA-avg | 0.703 | 0.711 | / | 0.719 | 0.720 | / | 0.716 | 0.719 | / | **0.731** | **0.737** | / |
| PLCO-BLCA | 0.648 | 0.642 | 0.224 | **0.718** | **0.718** | 0.013 | 0.679 | 0.681 | 0.040 | 0.687 | 0.692 | 0.032 |
| PLCO-BRCA | 0.601 | 0.597 | 0.040 | 0.706 | 0.703 | 2.32E-06 | 0.732 | 0.731 | 1.02E-07 | **0.756** | **0.751** | 1.85E-07 |
| PLCO-CRC | 0.791 | 0.812 | 4.32E-21 | 0.792 | **0.818** | 4.12E-22 | **0.795** | 0.813 | 8.87E-20 | 0.794 | 0.815 | 6.29E-23 |
| PLCO-LUAD | 0.617 | 0.660 | 0.030 | **0.699** | **0.755** | 3.36E-06 | 0.681 | 0.742 | 3.63E-06 | 0.672 | 0.721 | 7.27E-04 |
| PLCO-avg | 0.664 | 0.678 | / | **0.729** | **0.749** | / | 0.722 | 0.742 | / | 0.727 | 0.745 | / |
| CPTAC-GBM | **0.596** | **0.628** | 0.024 | 0.539 | 0.546 | 0.345 | 0.525 | 0.525 | 0.789 | 0.563 | 0.592 | 0.149 |
| CPTAC-LUSC | 0.703 | 0.723 | 2.75E-04 | 0.679 | 0.737 | 0.011 | 0.712 | 0.724 | 0.001 | **0.721** | **0.765** | 0.017 |
| CPTAC-PAAD | 0.526 | 0.532 | 0.487 | 0.531 | 0.537 | 0.466 | 0.535 | 0.539 | 0.086 | **0.560** | **0.576** | 0.102 |
| CPTAC-RCC | 0.747 | 0.757 | 3.26E-05 | 0.755 | 0.774 | 1.47E-05 | 0.740 | 0.753 | 4.95E-05 | **0.759** | **0.779** | 2.46E-05 |
| CPTAC-UCEC | 0.714 | 0.734 | 0.001 | 0.706 | 0.724 | 1.39E-04 | 0.681 | 0.705 | 0.015 | **0.717** | **0.745** | 0.009 |
| CPTAC-avg | 0.657 | 0.675 | / | 0.642 | 0.664 | / | 0.639 | 0.649 | / | **0.664** | **0.691** | / |

**REFERENCES**

1. Maron, O. & Lozano-Pérez, T. A framework for multiple-instance learning. *Advances in Neural Information Processing Systems* **10**, 570–576 (1998).

2. Ilse, M., Tomczak, J. & Welling, M. Attention-based deep multiple instance learning. In *International Conference on Machine Learning*, 2127–2136 (PMLR, 2018).

3. Wang, X. *et al.* Retccl: Clustering-guided contrastive learning for whole-slide image retrieval. *Med. Image Anal.* **83**, 102645 (2023).

4. Chen, R. J. *et al.* Towards a general-purpose foundation model for computational pathology. *Nat. Med.* **30**, 850–862 (2024).

5. Xu, H. *et al.* A whole-slide foundation model for digital pathology from real-world data. *Nature* **630**, 181–188 (2024).

6. Zimmermann, E. *et al.* Virchow2: Scaling self-supervised mixed magnification models in pathology. *arXiv preprint arXiv:2408.00738* (2024).

7. Cox, D. R. Regression models and life-tables. *J. R. Stat. Soc. B* **34**, 187–202 (1972).
